# Therapies for Mitochondrial Disease: Past, Present, and Future

**DOI:** 10.1002/jimd.70065

**Published:** 2025-07-25

**Authors:** Megan Ball, Nicole J. van Bergen, Alison G. Compton, David R. Thorburn, Shamima Rahman, John Christodoulou

**Affiliations:** ^1^ Brain and Mitochondrial Research Group Murdoch Children's Research Institute Melbourne Australia; ^2^ Department of Paediatrics University of Melbourne Melbourne Australia; ^3^ Royal Children's Hospital Melbourne Australia; ^4^ Victorian Clinical Genetics Services Murdoch Children's Research Institute Melbourne Australia; ^5^ Metabolic Unit Great Ormond Street Hospital London UK; ^6^ Mitochondrial Research Group UCL Great Ormond Street Institute of Child Health London UK

**Keywords:** clinical trials, gene therapy, mitochondrial disease, small molecule therapy, treatment

## Abstract

Mitochondrial disease is a diverse group of clinically and genetically complex disorders caused by pathogenic variants in nuclear or mitochondrial DNA‐encoded genes that disrupt mitochondrial energy production or other important mitochondrial pathways. Mitochondrial disease can present with a wide spectrum of clinical features and can often be difficult to recognize. These conditions can be devastating; however, for the majority, there is no targeted treatment. In the last 60 years, mitochondrial medicine has experienced significant evolution, moving from the pre‐molecular era to the Age of Genomics in which considerable gene discovery and advancement in our understanding of the pathophysiology of mitochondrial disease have been made. In the last decade, in response to the urgent need for effective treatments, a wide range of emerging therapies have been developed, driven by innovative approaches addressing both the genetic and cellular mechanisms underpinning the diseases. Emerging therapies include dietary intervention, small molecule therapies aimed to restore mitochondrial function, stem cell or liver transplantation, and gene or RNA‐based therapies. However, despite these advances, translation to clinical practice is complicated by the sheer genetic and clinical complexity of mitochondrial disease, difficulty in efficient and precise delivery of therapies to affected tissues, rarity of individual genetic conditions, lack of reliable biomarkers and clinically relevant outcome measures, and the dearth of natural history data. This review examines the latest developments in the pursuit to identify effective treatments for mitochondrial disease and discusses the barriers impeding their success in translation to clinical practice. While treatment for mitochondrial disease may be on the horizon, many challenges must be addressed before it can become a reality.

## 1 Introduction

Mitochondrial disease (MD) is a diverse group of disorders that disrupt mitochondrial energy generation by directly affecting oxidative phosphorylation (OXPHOS) or altering other essential mitochondrial pathways [1, 2]. OXPHOS occurs in the inner mitochondrial membrane, where electron transport in the respiratory chain is coupled with ATP production. OXPHOS was first described in 1961 [3] followed by the first clinical description of mitochondrial dysfunction in Luft disease the following year [4]. Mitochondria are integral to cellular metabolism and are critical for calcium buffering, lipid metabolism, maintaining cellular ion homeostasis, formation of reactive oxygen species (ROS) and integrating signalling pathways that govern cell survival and apoptosis [5]. Mitochondria adapt to cellular energy demands through dynamic processes such as fusion, fission, biogenesis (increasing mitochondrial mass) and mitophagy (mitochondrial quality control by selectively removing dysfunctional mitochondria from the cell) [6]. The biology underpinning mitochondrial function has been extensively reviewed elsewhere [5, 7, 8].

The minimum birth prevalence of MD is estimated to be 1 in 5000 [9] with a minimum point prevalence of 12.5 per 100 000 in adults, so most individual MDs are exceedingly rare [10]. MD can manifest at any point in life, is highly variable with a range of clinical signs and symptoms, predominantly affecting organs with a high energy demand, particularly the heart, muscle, and brain. MD is caused by pathogenic variants in nuclear encoded genes or mitochondrial DNA (mtDNA). The human mtDNA is a double‐stranded, circular DNA encoding 13 protein subunits of the respiratory chain, two ribosomal RNAs, and the complete set of 22 transfer RNAs required for mitochondrial protein synthesis [11]. The pathogenic role of mtDNA variants in human disease was established in 1988 with the identification of large‐scale deletions in individuals with myopathy and a maternally inherited mtDNA single nucleotide variant in families with Leber hereditary optic neuropathy (LHON) [12, 13]. Mitochondrial medicine has advanced considerably in the last 60 years, with the discovery of over 400 nuclear‐encoded genes implicated in MD [14] and the enhanced understanding of pathological mechanisms underpinning disease paving the way for the development of therapeutic strategies for MD.

There are unique challenges to successfully identifying therapeutic strategies for MD. The translation of potential therapies from preclinical studies to clinical practice has been hampered by the genetic and phenotypic complexity of MD, challenges in delivery of therapies into the mitochondria, lack of reliable biomarkers and outcome measures or well‐described natural history and the difficulty of recapitulating the pre‐clinical findings from animal models to human disease. Given the rarity of individual MDs, novel therapeutic strategies have often been described in small case reports or open‐label studies which are difficult to interpret given the inherent bias and confounding factors involved with such studies.

## 2 Current Management Approaches

For most primary MD, there is no currently available targeted therapy. Management is focused on symptoms and surveillance for the associated complications of MD.

### 2.1 Treatable Disorders

For a few specific MDs (predominantly defects of cofactor metabolism), there are targeted treatments available with a spectrum of efficacy (Table 1).

**TABLE 1:** Mitochondrial diseases with targeted therapy available.

Disease (OMIM)	Gene/s	Treatment
Coenzyme Q_10_ deficiency^a^	*COQ2*, *COQ4*, *COQ5*, *COQ6*, *COQ7*, *COQ8A*, *COQ8B, COQ9*, *PDSS1*, *PDSS2*, and *ADCK2*	Coenzyme Q_10_ [15, 16, 17]
Biotin‐thiamine‐responsive basal ganglia disease (#607483)	*SLC19A3*	Biotin Thiamine [18]
SLC25A19‐related thiamine transporter deficiency (#613710)	*SLC25A19*	Thiamine [19]
Thiamine pyrophosphokinase deficiency (#614458)	*TPK1*	Thiamine [20]
Pyruvate dehydrogenase deficiency (#312170)	*PDHA1*	Thiamine [21]
Biotinidase deficiency (#609019)	*BTD*	Biotin [22]
Holocarboxylase synthetase deficiency (#253270)	*HLCS*	Biotin [23]
Multiple acyl‐CoA dehydrogenase deficiency (#231690)	*ETFA*, *ETFB*, and *ETFDH*	Riboflavin [24]
FAD Transporter Deficiency (#616839)	*SLC25A32*	Riboflavin [25]
FAD Synthase Deficiency (#255100)	*FLAD1*	Riboflavin [25]
Dihydrolipoamide dehydrogenase (E3) deficiency (#246900)	*DLD*	Riboflavin [26]
ACAD9 deficiency (#611126)	*ACAD9*	Riboflavin [27]

^a^ OMIM for individual Coenzyme Q^10^ deficiency genes; #607426, #616276, #619028, #614650, #614654, #612016, #615573, #614654, #614651, and #614652.

#### 2.1.1 Coenzyme Q_10_

Coenzyme Q_10_ (CoQ_10_) shuttles electrons in the mitochondrial respiratory chain and, in its reduced form, is an effective antioxidant buffering free electrons, restoring other antioxidants (vitamins E and C) and protecting cells from oxidative damage [28]. It is commonly prescribed in MD, but clinical trials have shown conflicting evidence for its benefit (NCT00432744) [29], except in primary CoQ_10_ deficiency where it has been shown to lead to favorable outcomes in some (< 50%) individuals, although variable response has been reported which may be contributed to by inadequate uptake across the blood‐brain barrier [15, 16, 17, 30, 31]. Individuals reported to have objective or subjective improvement include those with pathogenic variants in *COQ2*, *COQ4*, *COQ5*, *COQ6*, *COQ8A*, and *COQ8B*, with reversal of renal disease or improvement in neurological features such as ataxia. Modified precursors of the quinone ring of CoQ_10_ such as 2,4‐dihydroxybenzoic acid have shown some promise in pre‐clinical studies, but further work is warranted to determine if this is a better alternative strategy [32].

#### 2.1.2 Thiamine

Thiamine (B1) is converted to its active form thiamine pyrophosphate by the enzyme thiamine pyrophosphate kinase encoded by *TPK1* and subsequently acts as a cofactor for mitochondrial alpha‐ketoacid dehydrogenases. Defects in thiamine metabolism show variable responses to thiamine supplementation [19]. Thiamine supplementation in TPK1 deficiency resulted in significant improvement in clinical symptoms in 50% of individuals [20]. Early treatment with high‐dose biotin and thiamine in *SLC19A3* deficiency (thiamine transporter resulting in biotin‐thiamine responsive basal ganglia disease) is associated with rapid clinical improvement and excellent outcomes [18, 33], thought to be attributed to biotin influencing *SLC19A3* expression [34]. Some individuals with PDHC deficiency also have a good response to thiamine; however, this response is variable and may be related to specific pathogenic variants in the E1α subunit, impacting the binding of thiamine pyrophosphate [21].

#### 2.1.3 Biotin

Biotin (B7) is a cofactor for five carboxylases (of which four are in the mitochondria) that play roles in many metabolic pathways. Biotinidase is the enzyme responsible for recycling protein‐bound biotin into free biotin. Lifelong biotin supplementation for biotinidase deficiency is well tolerated and can prevent the development of the neurological and cutaneous phenotype [22]. Prompt biotin supplementation is also recommended in individuals with holocarboxylase synthetase deficiency, which is associated with a defect in the enzyme that binds biotin to the biotin‐dependent carboxylases, although their clinical improvement is not as robust as in biotinidase deficiency [23].

#### 2.1.4 Riboflavin

Riboflavin (B2) is the precursor of the electron carriers flavin adenine dinucleotide (FAD) and flavin mononucleotide (FMN), which serve as cofactors for respiratory chain complex I and II, plus other mitochondrial dehydrogenases involved in multiple mitochondrial processes [25, 35]. Riboflavin has a positive effect on clinical symptoms in individuals with dihydrolipoamide dehydrogenase (E3) deficiency [26], FAD synthase and transporter deficiencies [25] plus respiratory chain complex I disorders, specifically ACAD9 deficiency [27, 36]. A large multinational cohort study of individuals with ACAD9 deficiency found that 65% had a positive clinical response to riboflavin supplementation [27]. High‐dose riboflavin improved clinical symptoms and biochemical abnormalities in most individuals with late‐onset multiple acyl‐coA dehydrogenase deficiency (MADD) and should be considered in all these individuals [24].

### 2.2 Nutritional Supplements

The use of nutritional supplements (vitamins, cofactors and antioxidants) for MD is widespread despite the dearth of high‐quality evidence for their efficacy in the majority of MD. Use of these supplements is widely disparate, ranging from single supplements to a range of nutritional supplements, often referred to as a “mitochondrial cocktail” [37]. These supplements have been used on a theoretical basis due to their potential impact on mitochondrial physiology. Examples include strategies to reduce oxidative stress (CoQ_10_, vitamin E, alpha lipoic acid, N‐acetyl cysteine) or provide deficient vitamins or cofactors (thiamine, riboflavin, folinic acid, and biotin). These supplements are generally regarded as low risk, with few reported adverse effects. Large clinical trials for these supplements have not occurred for several reasons, including the ease of access. In 2012, a Cochrane review found that there was no clear evidence supporting the use of these vitamin and cofactor supplements in MD [29]. A review of these supplements was published in 2020 [38].

### 2.3 Exercise

Individuals with MD often experience fatigue, exercise intolerance, and weakness. They may be less physically active, which can lead to muscle deconditioning and worsening exercise intolerance. Exercise training in individuals with MD has not only confirmed the physiological adaptations of stimulating mitochondrial biogenesis [39] and improving respiratory chain complex formation [40], but has also translated into clinically relevant improvement in muscle strength, exercise endurance, and quality of life [41]. Consensus recommendations from the Mitochondrial Medicine Society advise that endurance and resistance training are safe when instituted in a supervised, progressive manner beginning with low intensity and short duration activity in those that are physically able [42].

## 3 Emerging Therapies

**FIGURE 1:**
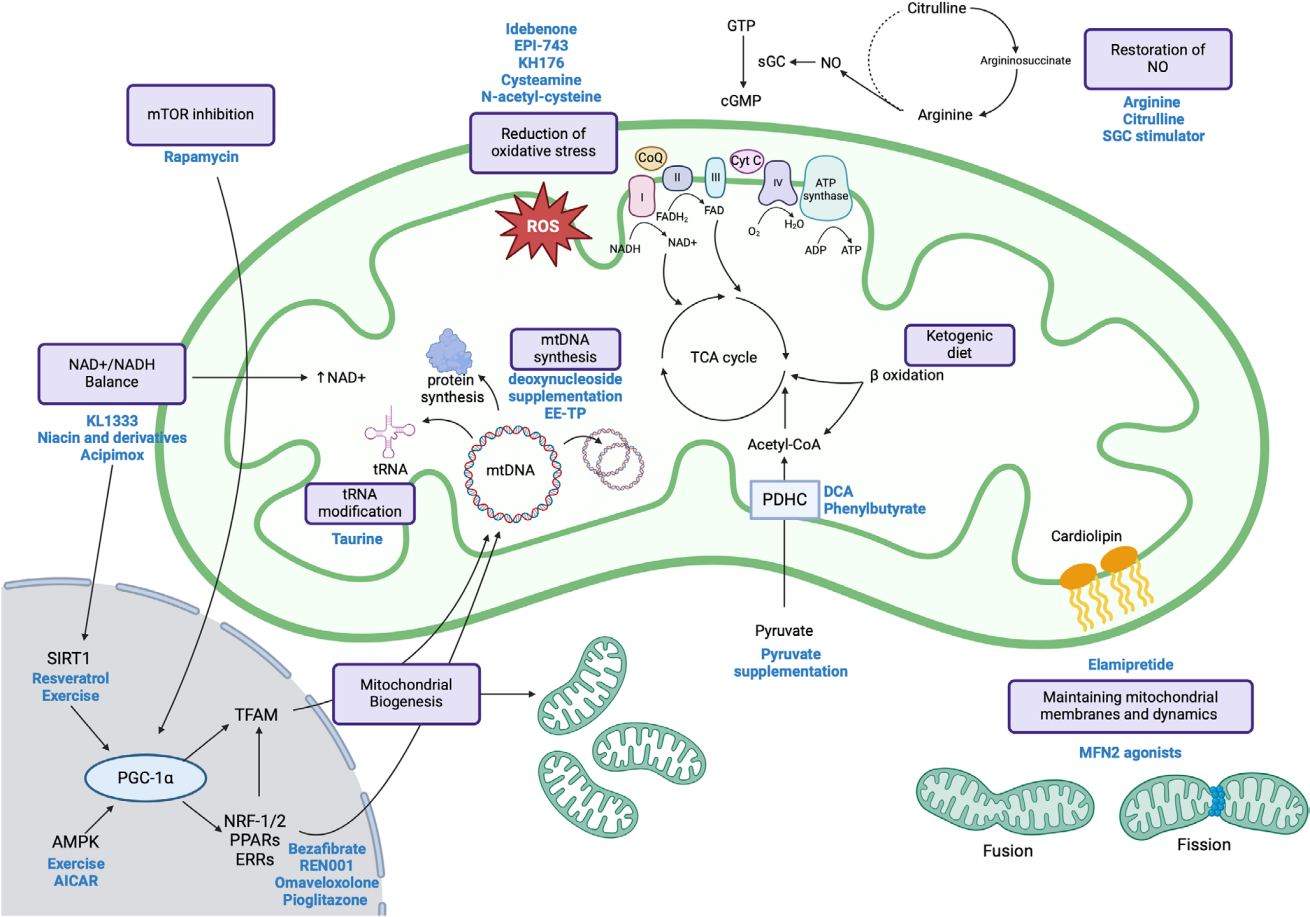
Emerging therapies for mitochondrial disease and their mechanism of action. This illustration outlines the different mechanistic targets for treatment of mitochondrial disease. The mitochondria are shown in green, and the nucleus is shown in grey. Therapeutic approaches are represented by a purple rectangle. Specific potential therapies are highlighted in blue adjacent to the therapeutic approach. Detailed mechanisms of action can be found in relevant sections of the text. ADP, adenosine diphosphate; AMPK, AMP activated protein kinase; ATP, adenosine triphosphate; cGMP, cyclic guanosine monophosphate; CoQ, Coenzyme Q_10_; Cyt *c*, cytochrome C; ERR, estrogen related receptor; FAD, flavin adenine dinucleotide; GTP, Guanosine triphosphate; mtDNA, mitochondrial DNA; NAD, nicotinamide adenine dinucleotide; NO, nitric oxide; NRF, nuclear respiratory factor; PDHC, pyruvate dehydrogenase complex; PGC‐1α, peroxisome proliferator‐activated receptor gamma coactivator 1‐alpha; PPAR, peroxisome proliferator‐activated receptor; ROS, reactive oxygen species; sGC, soluble guanylate cyclase; SIRT1, sirtuin 1; TCA, tricarboxylic acid; TFAM, transcription factor A, mitochondrial; tRNA, transfer RNA.Created in BioRender. Christodoulou, J. (2025) https://BioRender.com/r23l079.

Numerous pharmacological and non‐pharmacological strategies proposed for the treatment of MD are being developed with many active clinical trials underway (Table 2) targeting a wide range of mechanisms (Figure 1). Detailed references for each therapy and current clinical trial numbers are listed in Table S1.

**TABLE 2:** Active clinical trials of emerging therapies for mitochondrial disease.

Trial	Therapy	Disease	Trial Phase	Design	Age (yrs)	Primary outcome measure	Status
NCT06013397	Ketogenic diet	MELAS	N/A	Open‐label, single group assignment	Adults and children	NMDAS, biochemical indicators, HAMA, HAMD, WISC, MMSE, and cranial MRI	Not yet recruiting
NCT06340685	Triheptanoin	PDHC deficiency	Phase I	Open‐label, proof‐of concept study	1–18	Side‐effects related to GI distress, normalisation of biochemical markers of disease, more efficacious seizure, metabolic and disease control	Recruiting
NCT06644534	TTI‐0102	MELAS	Phase II	Multi‐centre, randomised, double‐blind, placebo‐controlled	16–60	Change in functional capacity (12MWT), incidence of treatment‐emergent adverse events (adverse events)	Not yet recruiting
NCT05241262	N‐acetylcysteine	m.3243A>G and low brain glutathione levels	Phase I	Open‐label, single group assignment, dose‐escalation	18–80	Maximum tolerated dose	Recruiting
NCT05218655	EPI‐743	PMD	Phase III	Open‐label, safety study for participants who had prior exposure in another clinical study	Adults and children	Adverse events	Enrolling by invitation
NCT04846036 (KHENERGYC)	KH176	PMD	Phase II	Randomised, placebo controlled, double‐blind	0–17	GMFM‐88	Active, not recruiting
NCT06451757 (KHENERFIN)	KH176	m.3243A>G	Phase III	Randomised, double‐blind, placebo controlled, parallel group	> 18	Neuro‐QoL Fatigue Short Form, 5XSST	Not yet recruiting
NCT05590468	Nicotinamide Riboside	MM	Phase II	Randomised, double‐blind, placebo‐controlled	> 18	6MWT	Recruiting
NCT06007391	Nicotinamide	OPA1 associated ADOA/+	Phase II/III	Open‐label, single group assignment	> 18	Adverse events	Not yet recruiting
NCT05650229 (FALCON)	KL1333	mtDNA disease	Phase II	Randomised, double‐blind, parallel‐group, placebo‐controlled, flexible‐dose	> 18	PROMIS Fatigue PMD Short Form, 30 s sit‐to‐stand test	Active, not recruiting
NCT06402123 (PRIZM)	Zagociguat	MELAS	Phase IIb	Randomised, double‐blind, placebo‐controlled, crossover	18–75	PROMIS Fatigue MELAS short form score, memory composite scores, international digit symbol substitution test scores, and adverse events	Recruiting
NCT05162768 (nuPower)	Elamipretide	MM (nuclear‐encoded)	Phase III	Randomised, double blind, parallel group, placebo‐controlled	18–70	6MWT	Active, not recruiting
NCT06017869	MNV‐201	Pearson Syndrome	Phase I	Open‐label, single dose study	1–18	Adverse events	Recruiting
NCT05962333	MAB	MM, m.3243A>G	Phase II	Open‐label, single group assignment, intra‐subjected controlled	18–64	Muscle strength and fatigue in treated biceps brachii compared to the untreated biceps brachii, adverse events, blood flow, and neurological vital signs	Recruiting
NCT04802707	Deoxycytidine and Deoxythymidine	Mitochondrial depletion syndrome	Phase II	Single‐arm, open‐label	0–60	Responder rate (EEG improvement, decreased seizure frequency, cognitive improvement, caregiver impression of improvement, clinical improvement, normal organics and metabolism functions)	Recruiting
NCT03845712	Deoxycytidine and Deoxythymidine	TK2 Deficiency	Phase II	Open‐label, safety and efficacy	Adults and children	Adverse events, laboratory measurements, and electrocardiograms	Active, not recruiting
NCT03639701	Thymidine and deoxycytidine	TK2 Deficiency	Phase I/II	Open‐label, single group assignment	Adults and children	Safety (laboratory markers, electrocardiogram, and diarrhoea)	Active, not recruiting
NCT02616484	DCA	PDHC deficiency	Phase III	Randomised, double‐blind, placebo‐controlled, crossover	0.5–17	ObsRO measure of health and adverse events	Active, not recruiting
NCT05972954 (PMD‐OPTION)	OMT‐28	mtDNA disease with clinically relevant myopathy and/or cardiomyopathy	Phase IIa	Open‐label, single‐arm, multiple‐phase, multi‐centre	18–60	Responder rate (difference in GDF15), adverse events	Active, not recruiting
NCT06292182	Near‐infrared light	PMD with ptosis	N/A	Open‐label, randomised, sequential assignment	3–18	Palpebral fissure width	Recruiting
NCT05293626	NR082	LHON, m.11778G>A	Phase I/II	Single‐arm, open‐label, dose‐finding	18–75	Adverse events and dose‐limiting toxicities	Active, not recruiting
NCT04912843	NR082	LHON, m.11778G>A	Phase II/III	Multi‐centre, randomised, double‐blind, sham‐controlled	18–75	Adverse events and dose limiting toxicities and BCVA	Recruiting
NCT03153293	GS010	LHON, m.11778G>A	Phase II/III	Open‐label, single group assignment	10–65	BCVA and computerised visual field	Active, not recruiting
NCT02161380	scAAV2‐CAG‐P1‐hND4v2	LHON	Phase I	Open‐label, dose‐escalation	> 15	Adverse events	Active, not recruiting
NCT06461286	PYC‐001	OPA1 associated ADOA	Phase Ia	Open‐label, single ascending dose study	> 18	Adverse events, change from baseline for vital signs measurements, 12‐lead ECG and clinical laboratory results	Recruiting

Abbreviations: ADOA, autosomal dominant optic atrophy; BCVA, best corrected visual acuity; ECG, electrocardiogram; EEG, electroencephalogram; GI, gastrointestinal; GMFM‐88, gross motor function measure‐88; HAMA, Hamilton anxiety scale; HAMD, Hamilton depression scale; LHON, Leber hereditary optic neuropathy; MELAS, mitochondrial encephalomyopathy with lactic acidosis and stroke‐like episodes; MM, mitochondrial myopathy; MMSE, mini‐mental state examination; MRI, magnetic resonance imaging; mtDNA, mitochondrial DNA; NMDAS, Newcastle mitochondrial disease adult scale; ObsRO, observed reported outcome; PDHC, pyruvate dehydrogenase complex; PMD, primary mitochondrial disease; PROMIS, patient‐reported outcomes measurement information system; WISC, Wechsler intelligence scale; 12MWT, 12‐min walk test; 5XSST, five times sit‐to‐stand test; 6MWT, 6‐min walk test.

### 3.1 Dietary Intervention

The ketogenic diet is a high fat, low carbohydrate diet that increases the production of ketone bodies by stimulating mitochondrial β‐oxidation of fatty acids. Ketone bodies are used as an alternate energy source for different tissues and can subsequently enter the tricarboxylic acid cycle via acetyl‐CoA. Other proposed effects of the ketogenic diet include stimulation of mitochondrial biogenesis [43, 44] and increased expression of genes involved in OXPHOS [45].

Ketogenic diet is emerging as standard practice in the management of PDHC deficiency [46, 47]. While multiple models of MD have reported benefits of a ketogenic diet (Table S1), the evidence base for ketogenic diet in humans with other MDs is inconclusive [48, 49]. Several case reports and observational studies have reported some benefit of ketogenic diet in MD [50, 51, 52]. Many clinical trials have shown that the ketogenic diet is effective in the treatment of non‐disease‐specific intractable epilepsy. A prospective open‐label, controlled study of ketogenic diet in 33 individuals with MD found that it was well tolerated and reduced seizure frequency in 76%, with the most significant improvement in individuals with mitochondrial encephalomyopathy with lactic acidosis and stroke‐like episodes (MELAS) or other mtDNA disease [53]. In contrast, five individuals with mitochondrial myopathy (MM) with single or multiple deletions were treated with a ketogenic diet and subsequently developed rhabdomyolysis within 2 weeks, resulting in termination of the study [54]. However, 2 years after the study, individuals were reported to have improved muscle strength, proposed to be secondary to muscle regeneration [54]. A feasibility study of the use of a Modified Atkins Diet in 20 individuals with MM reported that only eight individuals completed the 12‐week intervention, with five individuals having a mtDNA deletion experiencing muscle‐related adverse events [55]. A systematic review has suggested that a ketogenic diet should be considered in individuals with MD who have refractory epilepsy (except those with mtDNA deletion‐related myopathy and pyruvate carboxylase deficiency) [48].

Decanoic acid is a component of the medium chain triglyceride ketogenic diet and has been shown to stimulate mitochondrial biogenesis and decrease oxidative stress [43, 44]. An open‐label trial of K.Vita, a medical food containing a unique ratio of decanoic acid to octanoic acid, was shown to improve seizure frequency in individuals with drug‐resistant epilepsy, including those with MD [56]. Triheptanoin, a medium‐chain triglyceride of three 7‐carbon fatty acids, can be metabolized to provide two substrates (acetyl‐CoA and succinyl‐CoA) for the TCA cycle [57]. A phase I trial of triheptanoin in PDHC deficiency is currently recruiting (NCT06340685).

Other dietary interventions considered for MD include low residue diet and protein/valine restricted diets for ECHS1 and HIBCH deficiency. Low residue diet has been proposed to ameliorate the symptoms of gastrointestinal dysmotility, which is a common feature in MD. Low residue diet was shown to reduce gastrointestinal symptoms and laxative use in a phase II clinical trial in 28 adults with mtDNA‐related MD [58] with no evidence for its use in children thus far. Protein and valine restricted diets have been reported to be beneficial in some (but not all) individuals with *ECHS1* and *HIBCH* associated MD due to their involvement in the valine degradation pathway [59]. Valine restriction has also been shown to prolong survival in a *Drosophila* model of ECHS1 deficiency [60].

### 3.2 Manipulating Mitochondrial Biogenesis

Proliferation of mitochondria is a compensatory adaptation stimulated by caloric restriction, cold exposure, and exercise, to optimize OXPHOS. Pharmacological approaches aim to capitalize on the signaling pathways that regulate mitochondrial biogenesis, which are centered around the transcriptional coactivator peroxisome proliferator‐activated receptor (PPAR) gamma coactivator 1‐alpha (PGC‐1α) [61], which interacts with other transcription factors including PPARs and nuclear respiratory factors (NRFs) to control the expression of nuclear‐encoded mitochondrial genes. PGC‐1α is post‐translationally activated by AMPK and Sirtuin1 (Sirt1), which are themselves regulated by NAD^+^/NADH balance [61].

Bezafibrate is a pan‐PPAR agonist; it has an established safety profile in humans as it has been used in the treatment of dyslipidaemias. It increases the activity of OXPHOS in cultured patient cells [62, 63], although a mild increase in ROS was also noted in *DNM1L* patient fibroblasts [63]. Bezafibrate has had conflicting results in mouse models of MD, in part due to rodent‐specific hepatotoxicity (Table S1). A phase II, open‐label, non‐randomized trial in six adult patients with m.3243A>G‐related MM demonstrated that the administration of bezafibrate over 12 weeks resulted in a reduction of respiratory chain complex IV deficient muscle fibres and improved cardiac function, although no change in exercise capacity was reported (NCT02398201) [64]. Conversely, there was an increase in serum biomarkers of MD, including FGF21 and GDF15, with alterations in amino acids and TCA intermediates highlighting the importance of exploring long‐term outcomes.

REN001 is a PPAR‐δ agonist also proposed to induce mitochondrial biogenesis and improve fatty acid oxidation [65]. It is well‐tolerated (NCT03862846) but an international, multi‐centre phase II randomised, placebo‐controlled trial investigating its use in adults with MM failed to meet its primary (change in distance with 12‐min walk test (12MWT)) and secondary (change in FACIT‐fatigue scores) efficacy endpoints (NCT04535609). A phase II/III clinical trial in MM using another PPAR‐δ agonist, ASP0367, was terminated when it failed to meet the pre‐specified criteria for efficacy (NCT04641962).

Omaveloxolone is an oleanolic triterpenoid that influences mitochondrial biogenesis by preventing NRF2 degradation [66]. Omaveloxolone was well tolerated by adults with MM in a phase II randomized, double‐blind placebo‐controlled trial but did not alter either peak workload in exercise testing or distance traveled during a six‐minute walk test (6MWT) [67]. Despite this, lower heart rates and blood lactate levels were noted during the submaximal exercise test at 12 weeks [67].

Resveratrol is a naturally occurring polyphenol that is reported to induce mitochondrial biogenesis predominantly through activation of SIRT1 [68]. While preclinical studies in patient fibroblasts were promising, a randomized, double‐blind, placebo‐controlled, cross‐over study of resveratrol supplementation in patients with MMs or fatty acid oxidation disorders did not meet its primary or secondary endpoints, with the authors concluding that it did not improve exercise capacity in adults with MM [69].

Other therapies proposed to stimulate mitochondrial biogenesis include 5‐aminoimidazole‐4‐carboxamide ribonucleoside, epicatechin, and pioglitazone, which have yet to progress to clinical studies in MD (Table S1).

### 3.3 Restoration of NAD ^+^/NADH Balance

The vitamin B3 family contains niacin (also interchangeably termed nicotinic acid and vitamin B3) and its derivatives including nicotinamide riboside (NR), nicotinamide mononucleotide (NMN) and nicotinamide (also called niacinamide). These are processed by mitochondria to NAD^+^, which participates as a key metabolic intermediate for mitochondrial energy production [70].

Disruption of mitochondrial NAD^+^ homeostasis is a critical driver of MM pathogenesis in humans. Systemic NAD^+^ deficiency was reported in five adults with MM in an open‐label clinical trial. Blood and muscle NAD^+^ was corrected to the level of controls following niacin supplementation, with increased muscle strength and mitochondrial biogenesis also reported [71]. Some noncompliance is reported in individuals using nicotinic acid, as it causes flushing [72].

NR has been examined in multiple mouse models of MD, resulting in increased mitochondrial biogenesis and motor performance [73, 74]. An open label trial of NR in adults with MD with m.3243A>G mtDNA variant or single mtDNA deletions has been completed with no results published (NCT03432871). NMN was shown to increase NAD^+^ levels in skeletal muscle and improve the lifespan of a *Ndufs4* ^−/−^ mouse [75] but has not yet progressed to trials in humans with MD.

KL1333 acts as a NAD^+^ precursor and has been shown in MELAS patient fibroblast cells to increase intracellular NAD^+^ levels via NADH oxidation, increasing ATP levels, mitochondrial mass, and oxidative capacity [76]. A first‐in‐human study assessing the safety and tolerability of KL1333 and a phase Ia/Ib randomized, double‐blind, placebo‐controlled trial in primary MD found that it was well tolerated, with dose‐dependent gastrointestinal side effects, and suggested that KL133 improved fatigue, functional strength, and endurance [77].

Acimipox is a niacin derivative that is used for the treatment of hyperlipidaemia in diabetes mellitus. Acimipox has been shown to increase the expression of nuclear‐encoded OXPHOS genes and increase muscle ATP content and respiratory capacity in ex vivo human skeletal muscle [78]. A randomised double‐blind, placebo‐controlled trial examining the change in ATP content in skeletal muscle in adults with MM following 12 weeks of acipimox has been completed, with results yet to be published (ISRCTN12895613).

NADH and NADPH can be damaged to generate a toxic form, NAD(P)HX, which blocks many cellular processes and dehydrogenase enzymes and disrupts NAD^+^ balance. Two highly conserved metabolite repair enzymes, NAD(P)HX dehydratase (NAXD) and NAD(P)HX epimerase (NAXE) are involved in NAD(P)HX repair. Pathogenic variants in both *NAXD* [79] and *NAXE* [80] lead to a severe and lethal pediatric neurodegenerative disease that evolves rapidly following an episode of otherwise benign mild fever or infection in previously healthy children. A handful of NAXD [81, 82] and NAXE [83, 84, 85, 86, 87, 88] cases have been treated with niacin therapy at high doses, and this appeared to stall clinical regression.

### 3.4 Reducing Oxidative Stress

ROS generation is a normal byproduct of OXPHOS and plays a role in cellular signalling. ROS are scavenged by antioxidant enzymes in various cellular compartments. However, in dysfunctional mitochondria, excessive ROS production can outweigh the cellular antioxidant capacity, leading to depletion of antioxidants such as glutathione [89], resulting in cellular damage to lipids, proteins, mtDNA, and components of the respiratory chain. Multiple therapeutic agents have been developed to attempt to prevent the effects of pathological ROS production and accumulation and to reduce oxidative stress.

Idebenone can act as a potent antioxidant and electron shuttle in the respiratory chain, thus promoting ATP production and preventing oxidative damage by bypassing respiratory chain complex I. Studies of idebenone have predominantly occurred in LHON, and it has been conditionally approved for treatment in LHON in individuals 12 years and older by the European Medicines Agency. A randomised double‐blind, placebo‐controlled phase II study of individuals with LHON given idebenone treatment for 6 months failed to meet its primary endpoint (best recovery of visual acuity). However, on subgroup analysis, it was found that idebenone may have benefit for those individuals with discordant visual acuity [90]. Additionally, at follow‐up 30 months after this study, it was found that the beneficial effect of idebenone persisted in this subgroup despite discontinuation [91]. Clinically relevant recovery was observed in 46% of individuals with subacute/dynamic LHON in an expanded access program for long‐term treatment (mean 25.6 months) where longitudinal data were available [92]. Subsequently, an international, open‐label study in 199 individuals with LHON compared the use of idebenone over 24 months to an external natural history control cohort. It found favourable vision outcomes from idebenone, with the treatment effect varying depending on the disease phase and causative mtDNA variant, with the most consistent benefit seen in those individuals with the m.11778G>A variant [93]. These findings were supported by a meta‐analysis [94]. A phase II trial in 16 individuals with *OPA1‐*dominant optic atrophy found that best recovery of visual acuity improved following treatment with idebenone [95]. A phase IIa double‐blind, randomised, placebo‐controlled, dose‐finding study of idebenone for individuals with MELAS has been completed and did not meet the primary endpoint of change in cerebral lactate levels measured by magnetic resonance spectroscopy (MRS) (NCT00887562).

Vatiquinone (also known as PTC‐743 and EPI‐743) is another synthetic analogue of CoQ_10_, which can bind and modulate oxidoreductases. It has been reported to improve glutathione balance and redox status [96] and was suggested to suppress ferroptosis, an iron‐dependent form of cell death [97]. Vatiquinone has been evaluated in multiple small open‐label studies with initial promising results. However, the results from larger randomized, double‐blind studies are conflicting, with some failing to meet primary endpoints and others yet to be published. The first open‐label study of vatiquinone in 14 individuals with MD who were likely to progress to end‐of‐life care within 90 days found that over 90% of individuals had significant clinical improvement and quality of life [96]. Following this, a phase IIa, open‐label trial in 10 children with LSS found significant improvement in clinical outcome measures [98] with nine exhibiting arrested disease progression and/or reversal after 6 months of treatment. Another study examined the effect of vatiquinone in individuals with various MDs and found that there was an improvement in cerebellar uptake of ^99m^Tc labeled hexamethyl propylene amine oxime and clinical improvement in the Newcastle Paediatric Mitochondrial Disease Scale [99]. A small open‐label trial of vatiquinone in five individuals with LHON resulted in arrested disease progression and/or reversal of vision loss in four, with complete recovery of visual acuity in two [100]. A phase II randomized double‐blind placebo‐controlled clinical trial examining vatiquinone in children with LSS is yet to be published (NCT01721733/NCT02352896). However, a reduction in the number of individuals requiring hospitalization following treatment in comparison to those receiving placebo has been reported [101]. An open‐label study of vatiquinone in five children with RARS2 deficiency found that all children had a reduction in the frequency of their seizures [101]. In contrast, two double‐blind clinical trials for vatiquinone in individuals with MD failed to meet their primary endpoints (NCT01642056 and NCT04378075), following which a small open‐label trial in Pearson syndrome was terminated early due to the results of the other studies not supporting continuation (NCT02104336).

KH176 (Sonlicromanol) is an orally bioavailable derivative of vitamin E that exerts its antioxidant effect by targeting the thioredoxin system [102]. A Phase IIa randomized, double‐blind, placebo‐controlled, two‐way crossover trial (KHENERGY) of KH176 in 18 adults with *MT‐TL1* m.3243A>G related MD found that KH176 was well tolerated with no serious adverse events. While it failed to meet its primary outcomes (gait parameters), it did report positive effects on alertness and mood [103]. The phase IIb study (KHENERGYZE) included a randomized controlled dose‐selection study followed by a 52‐week open‐label extension study. It failed to meet its primary endpoint of change from placebo in attention domain score of cognitive functioning. However, positive effects were noted in other domains including mood, balance control, quality of life, pain, and fatigue [104].

Cysteamine is an approved treatment for cystinosis. Cysteamine breaks down cystine, forming cysteine‐cysteamine disulfide and cysteine, which is a precursor for the biosynthesis of the antioxidant glutathione [105]. Preclinical studies in models of MD suggested that cysteamine could reduce oxidative stress [106, 107] but an open‐label phase II trial examining delayed‐release cysteamine bitartrate (RP103) in 36 children with MD followed by a long‐term extension study was terminated due to lack of effect (NCT02023866 / NCT02473445). A multicenter, randomized, double‐blind, placebo‐controlled phase II trial of TTI‐0102, a precursor to cysteamine, in individuals with MELAS is currently active, but not yet recruiting (NCT06644534).

N‐acetylcysteine (NAC) is a precursor to the antioxidant glutathione that is generally well tolerated and safe. NAC and/or L‐cysteine supplementation has been reported to improve survival in TRMU deficiency [108]. NAC has been used in combination with metronidazole in the treatment of *ETHE1‐*related MD in an attempt to buffer the hydrogen sulfide load, with some reported clinical improvements [109].

### 3.5 Restoration of Nitric Oxide

Nitric oxide (NO) is formed during the conversion of arginine to citrulline by nitric oxide synthase (NOS) in vascular endothelial cells. Citrulline can subsequently be converted back to arginine via other enzymes. NO plays an important role in the balance of vascular smooth muscle tone, stimulating vasodilation and maintenance of blood flow through the microvasculature [110]. NO deficiency can occur due to inhibition of NOSs, depletion of arginine and citrulline, or sequestration of NO [111, 112]. NO deficiency can result in decreased perfusion in the microvasculature of different tissues, which may contribute to features in MD such as stroke‐like episodes, myopathy, and headaches [110].

In children and adults with MELAS, L‐arginine and L‐citrulline supplementation have both been shown to increase NO production [112, 113], with L‐citrulline having a more pronounced effect. Reactive hyperaemic index, which is typically low in endothelial dysfunction, was measured in children and adolescents with MD and increased with L‐arginine and L‐citrulline supplementation [110]. The provision of L‐arginine in acute stroke‐like episodes for individuals with MELAS has been recommended in a consensus statement from the Mitochondrial Medicine Society [114], but not by another expert group [115]. Current clinical practice varies worldwide. A series of open label studies of intravenous L‐arginine administration during acute stroke‐like episodes and prophylactic L‐arginine supplementation in individuals with MELAS led to clinical improvement within the first 24 h of a stroke‐like episode and decreased the frequency of episodes [116, 117, 118]. However, a systematic review of 37 studies and case reports concluded that there is not enough high‐quality evidence to currently support this as treatment in MELAS in the acute phase or for prophylactic treatment [119], highlighting the need for robust clinical trials.

A retrospective analysis of the use of L‐arginine in children with other MDs found that of the nine individuals who received intravenous arginine during a stroke‐like episode there was a positive clinical response in 47% of episodes [120]. A randomised, cross‐over, open label clinical trial of L‐citrulline and L‐arginine in MD examining the reactive hyperaemic index has been completed, with results yet to be published (NCT02809170). NO exerts many of its functions via soluble guanylyl cyclase (SGC) receptors [121]. A phase IIb trial of zagociguat, a SGC stimulator in individuals with MELAS is currently recruiting (NCT06402123).

### 3.6 mTOR Inhibition and the Immune System

Mechanistic target of rapamycin (mTOR) is a key regulator of cellular homeostasis, including the regulation of mitophagy and the immune system [122]. Inhibition of mTOR by rapamycin was shown to prolong the lifespan of the *Ndufs4* ^*−/−*^ mouse model of LSS [123]. Subsequently, many other in vivo and cellular models of MD have supported this finding, with multiple downstream pathways including protein kinase C being implicated as a contributory mechanism (Table S1). mTOR inhibitors have been trialled in small numbers of individuals with MD with variable effect. Two children with MD were treated with everolimus (a rapamycin analogue) with conflicting results. One child was a girl with LSS secondary to a homozygous variant in *NDUFS4* who, following treatment, had clinical and neuroradiological improvement. In contrast, the other child who had MELAS did not show any improvement and continued to deteriorate [124]. Four additional individuals with MELAS were also treated with everolimus or rapamycin following kidney transplant, resulting in improvement in clinical parameters in all four individuals [125]. A phase II trial to assess the safety and tolerability of ABI‐009 (nanoparticle albumin bound sirolimus) in children with LSS has been terminated due to withdrawal of a corresponding Investigational New Drug application (NCT03747328).

Recent work in a *Ndufs4* ^−/−^ mouse model of LSS has provided more evidence for the beneficial effects of mTOR inhibition in modulating immune system dysregulation in the pathogenesis of MD [122]. *Ndufs4* ^−/−^ mice were treated with a colony‐stimulator factor 1 inhibitor to deplete leukocytes, which resulted in the rescue of both the central nervous system (CNS) and systemic disease phenotype [122]. There are numerous anecdotal reports in MD reporting the beneficial effect of other immune modulating therapies, for example, intravenous immunoglobulin and corticosteroids, but there have been no clinical trials to date [126]. A cohort study reviewing interferon signaling in a diverse group of individuals with MD reported that the expression of interferon stimulated genes was upregulated to levels similar to those in primary interferonopathies [127]. The expression of interferon stimulated genes has the potential to be a biomarker for MD but also a potential therapeutic target. JAK inhibitors have been utilized in type I interferonopathies [128] and have the potential to be used as a therapeutic option for individuals with MD and dysregulated interferon signaling, for example, *ATAD3A*‐related disease [129].

### 3.7 Maintaining Mitochondrial Membranes, Dynamics and Shape

Mitochondria undergo continuous cycles of fusion and fission to maintain mitochondrial shape and mass and to ensure a balanced composition of mitochondrial contents. This is regulated by the activity of pro‐fusion proteins (MFN1, MFN2, and OPA1) and pro‐fission proteins (DRP1 and FIS1) [130]. Overexpression of OPA1 has shown improvement in mitochondrial respiratory capacity by stabilizing respiratory chain supercomplex formation [131, 132] and has ameliorated the clinical phenotypes of different mouse models of MD [132, 133]. MFN2 activation via allosteric MFN2 agonists promoted mitochondrial fusion and normalized axonal mitochondrial trafficking in the *Mfn2* mouse model of Charcot–Marie‐Tooth type 2A [134, 135].

Elamipretide interacts with the phospholipid cardiolipin in the inner mitochondrial membrane, preserving the mitochondrial cristae structure and respiratory chain supercomplex formation, resulting in improved ATP production and reduced ROS formation in models of impaired mitochondrial function [136] (Table S1). Phase I/II clinical trials of elamipretide in MM showed promising results in improving exercise performance in the 6MWT, a reduction in reported fatigue and muscle symptoms, and was generally well tolerated [137, 138]. However, the phase III trial in MM failed to meet its primary endpoints after 24 weeks and thus was terminated prior to the extension phase [139]. TAZPOWER was a phase II/III, 28‐week, randomized, double blind, placebo‐controlled crossover trial of elamipretide in 12 individuals with Barth syndrome followed by a 168‐week open label extension, with eight individuals reaching the endpoint. Initial findings from the blind phase found that there was no significant change in distance in the 6MWT or in the Barth syndrome symptom assessment (BTHS‐SA) total fatigue score [140] despite improved metabolomic profiles [141]. However, at 168 weeks, there was significant improvement in 6MWT, BTHS‐SA total fatigue score, and left ventricular stroke volume [142]. Comparison to matched natural history controls supported the findings [143]. A case series of elamipretide use in children with MD reported that it was safe and well tolerated [144].

### 3.8 Restoring mtDNA Synthesis

Maintenance of mtDNA is reliant on nuclear‐encoded proteins that are involved in either the mtDNA replication machinery, mitochondrial dynamics, or maintenance of nucleotide pool balance. Thymidine kinase 2 (TK2) is a key enzyme involved in the mitochondrial nucleotide pool maintenance for mtDNA synthesis. Studies in a *Tk2* murine model have shown that deoxynucleoside supplementation bypasses the enzymatic block, restores mtDNA copy number, and improves clinical phenotypes [145, 146]. An international multicentre compassionate use program examined the use of nucleoside supplementation in 16 children and adults with TK2 deficiency [147]. Those with severe early onset disease had the most significant benefit from nucleoside supplementation with improved survival and motor function, although those with childhood and later onset disease still had some benefits, including stabilisation or improvement of their clinical features [147]. Similar strategies have been utilised in pre‐clinical models of other mtDNA maintenance disorders, such as DGUOK [148], RRM2B [149], and POLG [150] deficiency, although no studies in humans have been completed to date. A phase II open‐label trial of deoxynucleoside supplementation in individuals with mtDNA depletion syndromes is currently recruiting (NCT04802707) [151]. Interim data showed good tolerance and some improvement in Newcastle Mitochondrial Disease Scale scores [151]. However, the interpretation of these interim findings is limited given the clinical trial design.

Mitochondrial neurogastrointestinal encephalopathy (MNGIE) is caused by bi‐allelic loss of function *TYMP* variants resulting in a defective thymidine phosphorylase (TP) enzyme causing an accumulation of thymidine. Erythrocyte‐encapsulated TP (EE‐TP) is proposed as a potential bridging therapy for MNGIE. It involves the ex vivo encapsulation of the recombinant *Escherichia coli* enzyme TP in autologous erythrocytes, which are then infused back into the individual [152]. The TP then catalyzes the metabolism of the deoxyribonucleosides in the blood, which reduces the level of these metabolites not only in blood but also in other organs. EE‐TP has been used in individuals with MNGIE with reported improvement in clinical features such as muscle strength and weight gain [153]. A phase II open‐label trial examining the safety, tolerability, and efficacy of EE‐TP in MNGIE has recently been withdrawn (NCT03866954).

### 3.9 Solid Organ and Stem Cell Transplantation

Liver transplantation has been reported to have some success in MD with predominant liver involvement; however, it remains controversial given that liver transplant does not correct the neurological or extra‐hepatic disease that often accompanies liver involvement in these disorders [154]. Case reports and single‐centre experiences of individuals with MD [155], predominantly mtDNA maintenance disorders including DGUOK deficiency [156, 157], non‐Alpers *POLG*‐related disease [154, 158] and MPV17 deficiency [159] who have undergone liver transplant have mixed outcomes. Liver transplant for individuals with MPV17 deficiency has been reported to be effective only in late‐onset and mild phenotypes [159] with high mortality reported in children [160].

Consensus guidelines on the management of MNGIE have suggested considering either haematopoietic stem cell transplant (HSCT) or liver transplant as a permanent treatment option for replacing thymidine phosphorylase activity and that the decision would depend on multiple patient‐ and centre‐related factors and experience, given the high risk of morbidity and mortality especially with HSCT [161]. Liver transplant has also been performed in a small number of individuals with ethylmalonic encephalopathy [162] with the rationale that the liver can replace the defective ETHE1 enzyme, reducing the accumulation of toxic hydrogen sulfide.

### 3.10 Mitochondrial Augmentation Therapy

Mitochondrial augmentation therapy (MAT) involves enriching autologous CD34+ hematopoietic stem cells (HSCs) with isolated mitochondria derived from healthy donor white blood cells or placenta prior to reinfusing back into the affected individual. Recently, multiple approaches including bone marrow transplantation and administering isolated mitochondria from wild‐type mice or humans were reported to improve morbidity and mortality in an *Ndufs4* ^*−/−*^ LSS mouse model [163]. Small open‐label compassionate use studies have reported the use of MAT in single large scale mtDNA deletion syndromes (SLSMDS) with some individuals improving in some clinical parameters [164, 165, 166]. However, it is difficult to interpret these results given the small number of individuals and the inherent bias involved in open‐label studies. A phase I/II clinical trial to evaluate the safety and efficacy of MAT in children with Pearson syndrome has been completed with results yet to be published (NCT03384420). Treatment with autologous mesangioblasts (MABs) is currently being explored in a phase II trial (NCT05962333) in adults with m.3243A>G‐related MM given the tendency of MABs to have lower levels of variant mtDNA compared to skeletal muscle [167].

### 3.11 Other Therapeutic Approaches

Dichloroacetate (DCA) stimulates the activity of pyruvate dehydrogenase complex (PDHC) by inhibiting pyruvate dehydrogenase kinase (which inactivates PDHC). DCA has been shown to reduce postprandial or exercise blood lactate in individuals with congenital lactic acidosis and other MD; however, it did not result in improved clinical outcome measures [168, 169]. A double‐blind, placebo‐controlled, randomized clinical trial of DCA in individuals with MELAS was terminated due to worsening peripheral neuropathy in many individuals [170]. Phenylbutyrate, another inhibitor of pyruvate dehydrogenase kinase, has been shown to increase PDHC activity in vitro and in vivo [171, 172]. A phase II open‐label clinical trial investigating the safety and the effect of phenylbutyrate on blood lactate in individuals with PDHC is completed, with results yet to be published (NCT03734263).

Pyruvate supplementation has been proposed to rebalance NAD^+^/NADH levels in MD. While case reports and small case series have reported that pyruvate supplementation has a positive effect on biochemical markers and clinical outcomes in some individuals with MD, a systematic review of pyruvate supplementation concluded that there was little evidence to support pyruvate supplementation in PMD [173]. The combination of pyruvate and uridine ameliorated the OXPHOS defect in patient‐derived fibroblasts from a diverse range of MD and in an in vivo lethal rotenone model in zebrafish [174], highlighting the need for further studies.

Taurine is another potential option in the treatment of MELAS. It can modify taurine‐containing uridines of the anticodons of some mitochondrial tRNAs to enhance appropriate synthesis of mitochondrial proteins [175]. Other proposed mechanisms include maintenance of calcium concentration and decreasing superoxide generation [176]. Taurine supplementation in an open‐label trial of 10 individuals with MELAS significantly reduced the annual relapse rate of stroke‐like episodes. Sixty percent of individuals met the primary endpoint (complete prevention of stroke‐like episodes) [177].

OMT‐28 is a synthetic omega‐3 epoxyeicosanoid and has been proposed to prevent hypoxia/reoxygenation‐induced mitochondrial dysfunction and inflammasome activation in cultured cardiomyocytes and isolated mouse hearts exposed to stressors [178]. A phase IIa open‐label clinical trial in adults with mtDNA disease is currently underway (NCT05972954).

In vivo and in vitro studies have shown that chronic hypoxia (11% O_2_) can benefit mitochondrial function and can rescue the neurological phenotype of the *Ndufs4* ^*−/−*^ LSS mouse model [179]. Further work reported that moderate hypoxia or intermittent hypoxia did not have the same benefit [180]. The *Ndufs4* ^*−/−*^ mouse displayed impaired oxygen consumption and brain tissue hyperoxia. Strategies that normalised the brain hyperoxia (anaemia, carbon monoxide) rather than activating the hypoxia transcriptional response reversed the neurological phenotype. This led the authors to suggest that the unused oxygen may be a driver of pathology, so therapeutic strategies that decrease oxygen delivery may be protective [181]. These data are all pre‐clinical and need to be validated in further in vivo or human studies, which may be highly challenging.

Red light therapy is the use of low energy lasers to radiate tissue with light at near infrared (NIR) range (630–1000 nm). It has been suggested that NIR light therapy improves mitochondrial function via stimulation of cytochrome *c* oxidase, enhancing ATP production [182]. There are no published studies of NIR light therapy in individuals with MD. An open label trial of NIR therapy in four individuals with LHON examined the use of 630 nm wavelength laser emission to the closed eye for 80 s twice daily for 12 months but was terminated as they were unable to obtain the primary outcome measurements (NCT01389817). The Red Light in Mitochondrial Disease Study (REaLMS) is aiming to examine whether exposure to NIR light therapy improves the muscle function and mobility of individuals with MD due to the pathogenic m.3243A>G mtDNA variant.

## 4 Gene and Genetic Therapies

**FIGURE 2:**
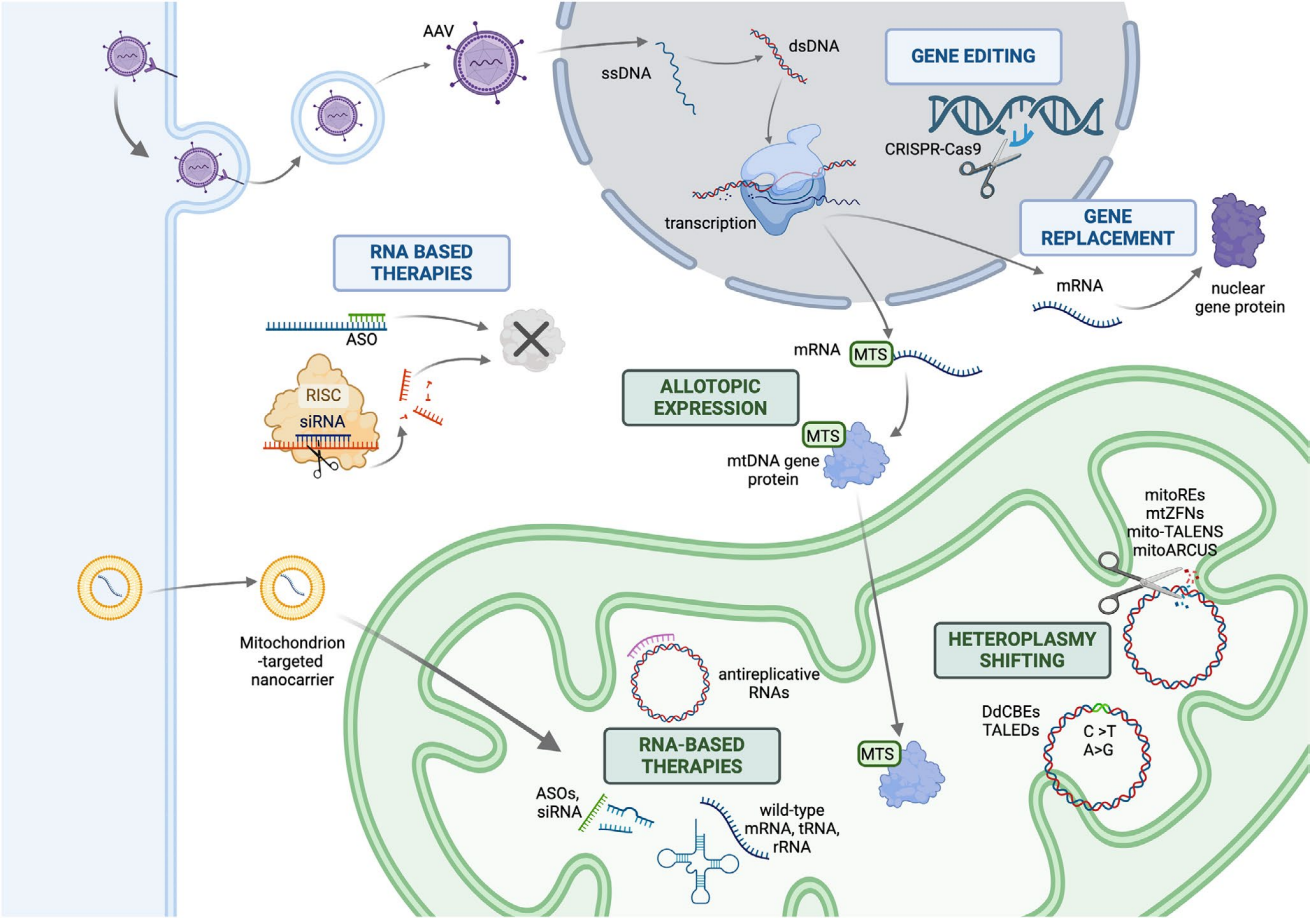
Gene and genetic therapies proposed as targeted treatments of mitochondrial disease. This schematic illustration outlines the different proposed gene therapies for treatment of mitochondrial disease. The mitochondria are shown in green, and the nucleus is shown in grey. Therapeutic approaches for nuclear gene‐associated disease are represented by blue rectangles. Therapeutic approaches for mtDNA gene‐associated disease are represented by green rectangles. Detailed mechanisms of action can be found in relevant sections of the text. AAV, adeno‐associated virus; ASO, antisense oligonucleotide; DdCBEs, DddA‐derived cytosine base editors; dsDNA, double‐stranded DNA; mitoARCUS, mitochondrial‐targeted ARCUS; mitoREs, mitochondrial‐targeted restriction endonucleases; mito‐TALENs, mitochondria‐targeted transcription activator‐like effector nucleases; mRNA, messenger RNA; MTS, mitochondrial targeting sequence; mtZFNs, mitochondrial‐targeted zinc finger nucleases; RISC, RNA‐induced silencing complex; rRNA, ribosomal RNA; siRNA, small interfering RNA; ssDNA, single‐stranded DNA; TALEDs, TALE‐linked deaminases; tRNA, transfer RNA.Created in BioRender. Christodoulou, J. (2025) https://BioRender.com/j08l091.

Gene therapy is still an emerging field for nearly all MD except LHON. There are several different approaches to gene therapy, including gene replacement therapy, RNA‐based therapy that can be used to either function similarly to gene replacement therapy or to silence the expression of genes, and gene or base editing shown in Figure 2 [183]. Gene therapy can be delivered in vivo (delivered directly into the individual) or ex vivo (gene therapy is delivered to relevant cells that have been removed from the individual which are then re‐infused following gene replacement or editing). Gene therapy can be delivered through non‐viral approaches, which may include chemical methods and utilizing endogenous mitochondrial import machinery, or more commonly in viral vectors, such as adeno‐associated virus vectors (AAVs) [183]. The approach to gene therapy in MD is dependent on whether the disease variant is in nuclear DNA or mtDNA.

### 4.1 Nuclear Gene‐Associated Mitochondrial Disease

#### 4.1.1 Gene Replacement

Most pre‐clinical studies of gene therapy in MD have utilized the viral vector recombinant AAVs (rAAVs) for gene replacement therapy. AAVs are small viruses that consist of a capsid and single‐stranded DNA. The AAV DNA can be replaced by a therapeutic transgene which, when released from the rAAV capsid in the nucleus of a cell, is converted into double‐stranded DNA, which is subsequently transcribed and translated, allowing for expression of the transgene. As they are episomic, they persist long‐term as extrachromosomal DNA and have a low risk of integrating into the host genome. However, this lack of integration can result in vector genome dilution in mitotically active tissues. There are multiple different naturally occurring AAV capsid serotypes that have been studied, all having different cell and tissue tropism [184].

rAAVs have been examined in over 12 mouse models of nuclear‐encoded MD, including *Ant1* [185], *Aifm1* [186], *Dguok* [187], *Ethe1* [188], *Mpv17* [189], *Ndufs4* [190, 191, 192], *Tymp* [193, 194, 195], *Opa1* [196], *Tafazzin* [197, 198], *Ndufs3* [199], *Slc25a46* [200], *Fdxr* [201], *Tk2* [202], and *Surf1* [203]. While results in mouse models have been promising, they have also highlighted the challenges in translating gene replacement therapy into clinical practice, with none progressing to clinical trials in humans to date.

Multi‐organ transduction by gene therapy would be essential in most MDs and is limited by the route of administration and AAV serotype tropism. Diseases with a predominant neurological phenotype require CNS transduction, which has only been achieved in mouse models with invasive routes such as intrathecal or intracerebroventricular, or with the AAV PHP.B vector, which does not transduce the human CNS as it does in the mouse models [199, 200]. This has necessitated the development of other delivery methods such as lipid‐based nanocarriers whose potential to traverse the blood brain barrier needs to be further explored [204]. The AAV vector genome is limited to a size of 4.7 kb, so full length transgenes larger than that would not be amenable to rAAV gene replacement therapy. Another challenge is that mouse models of MD do not always recapitulate the human phenotype, meaning that therapeutic effects can be difficult to interpret [203, 205]. Immune response to AAV vector‐based gene therapy, usually in the context of neutralising antibodies, can result in some individuals having a reduced response to treatment [206, 207]. Additionally, safety considerations have been raised with recent reported severe adverse effects in individuals with other monogenic conditions treated with intravenous high dose AAV‐based gene replacement therapy [208, 209].

#### 4.1.2 RNA‐Based Therapies

RNA‐based therapies can function either by providing the transgene in the form of mRNA or by modulating gene expression via antisense oligonucleotides (ASOs), small interfering RNAs (siRNAs), or microRNAs, amongst others. Autosomal dominant phenotypes that are secondary to gain‐of‐function or dominant‐negative effects may be amenable to ASOs or siRNAs. ASOs are short single‐stranded oligonucleotides that can affect gene expression via RNA degradation, inhibition of translation, or modulation of splicing [210]. ASOs were used to target *OPA1* mRNA in patient fibroblasts, promoting nonsense‐mediated decay of the nonproductive *OPA1* mRNA, increasing expression of the functional protein and improving mitochondrial respiratory chain function [211], leading to a first‐in‐human clinical trial that is currently recruiting (NCT06461286). Interestingly, ASOs targeting *DRP1* restored mitochondrial morphology in *Mfn2* ^*−/−*^ mouse embryonic fibroblasts [212]. siRNAs are small double‐stranded oligonucleotides that decrease or silence gene expression predominantly via RNA‐induced silencing complex. Targeted delivery of RNA‐based therapies has challenges, especially in MD. Whilst RNA‐based therapies have not yet progressed to clinical trials in MD, there are some that have had successful translation into clinical practice for other conditions, for example, nusinersen for the treatment of spinal muscular atrophy [213].

#### 4.1.3 Gene Editing

CRISPR‐Cas9 mediated gene editing has been used in a *Tymp* mouse model [214] and *OPA1* iPSC model [215], with success in demonstrating gene correction and restoration of mitochondrial homeostasis. The utility of CRISPR‐Cas9 is limited to nuclear genes given the inability of mitochondria to import the guide RNA upon which CRISPR‐Cas9 systems rely.

### 4.2 Manipulation of the Mitochondrial Genome

#### 4.2.1 Allotopic Expression

Allotopic expression is the expression of a mtDNA transgene in the nucleus with the translated protein subsequently imported into the mitochondria by the inclusion of a mitochondrial‐targeting sequence (MTS). It requires recoding to correct specific mtDNA codons that do not use the universal genetic code. The allotopic expression method was used to rescue mitochondrial respiratory chain function in cybrids containing the pathogenic *MT‐ATP6* m.8993T>G variant [216] and was subsequently shown to improve visual function and prevent retinal cell degeneration in a rat model of LHON [217]. The delivery of a *MT‐ND4* transgene in an AAV2 vector has been studied in individuals with LHON caused by the pathogenic m.11778G>A variant. Multiple clinical trials have shown that intravitreal injections of rAAV2‐*ND4* are associated with improved visual acuity [218, 219, 220, 221, 222, 223, 224, 225, 226, 227] and are generally well tolerated with minimal adverse effects [228]. Bilateral visual improvement after unilateral injection has been well reported. This unexpected finding in the contralateral eye has contributed to delays in approval given the difficulty of interpreting the effect of treatment without controls. A recent meta‐analysis has proposed that individuals with LHON who received intravitreal injections of AAV2 gene therapy (lenadogene nolparvovec) showed a better clinically relevant recovery rate compared to those treated with idebenone or those who were historically untreated [94].

#### 4.2.2 RNA‐Based Therapies

Several approaches to RNA‐based therapies have been proposed for mtDNA disorders, including transfecting wild‐type mRNA, tRNA, or rRNA into mitochondria to rescue mitochondrial respiratory function [229, 230], modulating mitochondrial gene expression via ASOs or siRNAs [231], and using antireplicative RNA molecules to shift heteroplasmy in cells [232, 233, 234]. To our knowledge, these therapies have only been examined in in vitro models and are yet to progress to in vivo models of MD. The main challenge is the delivery of these therapeutic agents into the mitochondria. Possible methods include direct import using a mitochondrion‐targeted carrier, such as the liposome‐based nanocarrier MITO‐porter [235]. However, further work is required to determine if these methods are effective in vivo.

#### 4.2.3 Heteroplasmy Shifting

Shifting heteroplasmy to favor wild type mtDNA is a potential therapeutic approach to mtDNA disease. Strategies postulated to achieve this include eliminating variant mtDNA by introducing double strand breaks (DSBs), base editing, and selectively stalling mtDNA replication using antireplicative machinery with either peptide nucleic acid oligomers [236] or antireplicative RNA molecules, as described above.

mtDNA rapidly degrades in response to DSBs, a mechanism that is exploited by the use of nucleases to attempt to shift heteroplasmy. Mitochondrially targeted restriction endonucleases (mitoREs) target the unique restriction sites associated with some pathogenic mtDNA variants to create site‐specific DSBs, resulting in mtDNA degradation and heteroplasmy shift [237, 238]. MitoREs are limited as not all pathogenic mtDNA variants are associated with unique restriction sites. Engineered programmable mitochondrially targeted nucleases, transcription activator‐like effector nucleases (mito‐TALENs) [239, 240, 241] and zinc finger nucleases (mtZFNs) [242, 243, 244, 245] have been shown to induce the elimination of variant mtDNA in in vitro and in vivo models of mtDNA disease. The ZFNs and TALENs direct the endonuclease to specific mtDNA sequences containing mtDNA point mutations or deletions. mtDNA targeted meganuclease (mitoARCUS) is a bacterial endonuclease that is engineered to create site‐specific DSBs. The use of mitoARCUS resulted in almost complete elimination of mutant mtDNA in human *MT‐TL1* m.3243A>G cybrid cells with associated improved mitochondrial respiration [246] and in a m.5024C>T mouse model [247]. While these strategies are promising for heteroplasmic variants and need to be further explored, they will not be applicable for homoplasmic variants as their use would most likely result in near complete elimination of mtDNA.

Base editing techniques have been proposed to introduce specific nucleotide changes into mtDNA without requiring DSBs and therefore to potentially shift heteroplasmy levels. Due to the difficulties importing RNAs into mitochondria, CRISPR‐free base editing strategies have been used to edit or introduce variants into the mtDNA genome in in vivo and in vitro models. These strategies include DddA‐derived cytosine base editors (DdCBEs) [248, 249, 250, 251, 252, 253, 254] or zinc finger DdCBEs (ZF‐DdCBEs) [255] for C‐to‐T transitions and TALEN‐linked deaminases(TALEDs) [256] for A‐to‐G transitions. Off‐target base editing has been reported [257] and strategies to prevent this are needed, as demonstrated in a recent study that showed promising clinical and biochemical improvement in a mouse model of mt‐tRNA(Ala) dysfunction [258].

### 4.3 Prevention of mtDNA Disorders

Given the current lack of treatment for MD, providing reproductive options for families to prevent transmission is important. For mtDNA disorders, determining the most appropriate reproductive option is difficult given the complexity of mtDNA inheritance and the lack of strong genotype–phenotype correlation for most variants. Prenatal diagnosis and preimplantation genetic testing may be considered for some women with low to moderate heteroplasmy levels but are not suited for those with high heteroplasmy or homoplasmy [259]. Studies exploring mitochondrial replacement therapy (MRT) are underway in the United Kingdom and Australia. MRT involves the removal of a nucleus from either a zygote (pronuclear transfer) or an oocyte (maternal spindle transfer), which is subsequently placed into an enucleated cell from a donor with normal mtDNA at the same embryonic stage [260].

## 5 Conclusions

The future of MD therapies holds promise, with advances in innovative approaches progressing in parallel with increasing knowledge of the pathophysiological mechanisms underlying MD. However, implementation of these therapeutic strategies still presents significant challenges. The genetic diversity of MD and complexity of mitochondrial physiology make developing a one‐size‐fits‐all approach difficult, and tailored, gene‐specific therapies require significant investment and infrastructure for development. Gene and RNA‐based therapies are considered one of the most promising avenues, offering the potential to directly address the underlying genetic defect. However, given the sheer number of genes (> 400) [14] currently associated with MD, translation to clinical practice is impeded by the rarity of each MD, availability of funding, complexity of development and implementation, and the requirement for targeted‐delivery systems to improve efficacy and mitigate off‐target effects.

The integration of genomic sequencing into standard clinical practice has allowed for the creation of large cohorts of individuals with genetically confirmed MD. This is essential for developing comprehensive natural history data and the adequately powered international clinical trials required to create an evidence base for emerging therapeutic strategies. Novel MD biomarkers such as interferon signaling genes or those identified by metabolomic strategies have the potential to be used as reliable measures of treatment efficacy. However, clinically meaningful outcome measures need to be further developed and validated in natural history cohorts to be useful for clinical trials. Overcoming these barriers will be critical in the development of personalized therapies to provide hope for those affected by MD.

## Author Contributions

Conceptualization and planning: M.B. and J.C. Writing – original Draft: M.B. Writing – review and editing: J.C., D.R.T., A.G.C., N.J.V.B. and S.R. Supervision: J.C., D.R.T., and A.G.C.

## Ethics Statement

The authors have nothing to report.

## Conflicts of Interest

The authors declare no conflicts of interest.

## Supporting information


**Data S1.** Supporting Information.
