# Supplementary material for: Therapies for Mitochondrial Disease: Past, Present, and Future
Source: J Inherit Metab Dis. 2025 Jul 25;48(4):e70065. doi: 10.1002/jimd.70065 (PMC12301291; doi:10.1002/jimd.70065)
Supplement: Supplementary file 1 — Data S1. Supporting Information. [file JIMD-48-0-s001.docx]

| **Therapeutic Strategy** | **Emerging Therapy** | **Relevant preclinical studies** | **Relevant Clinical Trials / Case Studies / Reviews** |
| --- | --- | --- | --- |
| Dietary Intervention | Ketogenic Diet | (1)  (2)  (3)  (4)  (5)  (6) | (7)  (8)  (9)  (10)  (11)  (12)  (13)  NCT06013397 |
|  | Decanoic Acid | (5)  (6) | (14). |
|  | Triheptanoin |  | NCT06340685 |
|  | Low Residue Diet |  | (15) |
| Manipulating mitochondrial biogenesis | Bezafibrate | (16)  (17)  (18)  (19)  (20)  (21) | (22)  NCT04561466 |
|  | REN001 | (23) | NCT03862846  NCT04535609  NCT05267574 |
|  | ASP0367 |  | (24)  NCT04641962 |
|  | Omaveloxolone | (25) | (26) |
|  | Resveratrol | (27)  (28)  (29)  (30) | (31) |
|  | 5-aminoimidazole-4-carboxamide ribonucleoside | (18)  (32)  (33)  (34)  (35) |  |
|  | Epicatechin | (36) |  |
|  | Pioglitazone | (37) |  |
| Restoration of NAD+/NADH balance | Niacin |  | (38)  NCT04538521 |
|  | Nicotinamide Riboside | (39)  (40)  (41)  (42)  (43) | (44)  NCT03432871  NCT05590468 |
|  | Nicotinamide Mononucleotide | (45) |  |
|  | Nicotinamide |  | NCT06007391 |
|  | KL1333 | (46) | (47).  NCT05650229 |
|  | Acimipox | (48) | ISRCTN12895613 |
| Reducing oxidative stress | Idebenone | (49) | (50)  (51)  (52)  (53)  (54)  (55)  (56)  (57)  NCT00887562 |
|  | EPI-743 | (58)  (59)  (60) | (61).  (62)  (63).  (64).  (65)  (66)  NCT01642056  NCT01721733/ NCT02352896  NCT02104336  NCT04378075  NCT05218655 |
|  | KH176 | (67)  (68)  (69) | (70)  (71)  NCT04846036  NCT06451757 |
|  | Cysteamine | (72)  (73) | NCT02023866 / NCT02473445 |
|  | TTI-0102 |  | NCT06644534 |
|  | N-acetylcysteine | (74)  (75)  (76)  (77)  (73)  (78) | (79)  (80)  (81)  (82)  (83)  NCT05241262 |
| Restoration of nitric oxide | Arginine +- Citrulline |  | (84)  (85)  (86)  (87)  (88)  (89)  (90)  (91)  (92)  (93)  (94)  NCT02809170  NCT03952234 |
|  | Zagociguat | (95) | NCT04475549  NCT06402123 |
| mTOR inhibition and the immune | Rapamycin and its analogues | (96)  (97)  (98)  (99)  (100)  (101)  (102)  (103)  (104) | (105)  (101)  NCT03747328 |
| Maintaining mitochondrial membranes, dynamics and shape | Elamipretide | (106)  (107)  (108)  (109)  (110)  (111)  (112)  (113)  (114)  (115) | (116, 117)  (116)  (117)  (118)  (119)  (120)  (121)  (122)  (123)  (124)  NCT05162768 |
| Restoring mtDNA synthesis | Deoxynucleoside supplementation | (125)  (126)  (127)  (128)  (129) | (130)  NCT04802707(131)  (132)  NCT03845712  NCT03639701 |
|  | Erythrocyte-encapsulated thymidine phosphorylase |  | (133)  (134)  NCT03866954 |
| Solid Organ and Stem cell transplantation | Solid Organ and Stem cell transplantation |  | (135)  (136)  (137)  (138)  (139)  (140)  (141)  (142)  (143)  (144)  (145) |
| Mitochondrial augmentation therapy | Mitochondrial augmentation therapy | (146)  (147)  (148)  (149) | (150)  (151)  (152)  NCT03384420 |
|  | Autologous mesangioblasts | (153) | NCT05962333 |
| Other | DCA | (154)  (155) | (156)  (157)  (158)  (159)  NCT02616484 |
|  | Phenylbutyrate | (160)  (161) | NCT03734263 |
|  | Pyruvate | (162) | (163) |
|  | Taurine | (164)  (165)  (166) | (167) |
|  | OMT-28 | (168) | NCT05972954 |
|  | Chronic Hypoxia | (169)  (170)  (171) |  |
|  | Red light therapy |  | NCT01389817  NCT06292182 |

**Supplementary Table 1:** Summary of relevant references for emerging therapies.

**References**

1. Santra S, Gilkerson RW, Davidson M, Schon EA. Ketogenic treatment reduces deleted mitochondrial DNAs in cultured human cells. Ann Neurol. 2004;56(5):662-9.

2. Ahola-Erkkilä S, Carroll CJ, Peltola-Mjösund K, Tulkki V, Mattila I, Seppänen-Laakso T, et al. Ketogenic diet slows down mitochondrial myopathy progression in mice. Hum Mol Genet. 2010;19(10):1974-84.

3. Schiff M, Bénit P, El-Khoury R, Schlemmer D, Benoist J-F, Rustin P. Mouse Studies to Shape Clinical Trials for Mitochondrial Diseases: High Fat Diet in Harlequin Mice. PLoS ONE. 2011;6(12):e28823.

4. Nunnari J, Suomalainen A. Mitochondria: in sickness and in health. Cell. 2012;148(6):1145-59.

5. Hughes SD, Kanabus M, Anderson G, Hargreaves IP, Rutherford T, O'Donnell M, et al. The ketogenic diet component decanoic acid increases mitochondrial citrate synthase and complex I activity in neuronal cells. J Neurochem. 2014;129(3):426-33.

6. Kanabus M, Fassone E, Hughes SD, Bilooei SF, Rutherford T, Donnell MO, et al. The pleiotropic effects of decanoic acid treatment on mitochondrial function in fibroblasts from patients with complex I deficient Leigh syndrome. J Inherit Metab Dis. 2016;39(3):415-26.

7. Lee YM, Kang HC, Lee JS, Kim SH, Kim EY, Lee SK, et al. Mitochondrial respiratory chain defects: underlying etiology in various epileptic conditions. Epilepsia. 2008;49(4):685-90.

8. Kang H-C, Lee Y-M, Kim H, Lee J, Slama A. Safe and Effective Use of the Ketogenic Diet in Children with Epilepsy and Mitochondrial Respiratory Chain Complex Defects. Epilepsia. 2007;48:82-8.

9. Martikainen MH, Päivärinta M, Jääskeläinen S, Majamaa K. Successful treatment of POLG-related mitochondrial epilepsy with antiepileptic drugs and low glycaemic index diet. Epileptic Disord. 2012;14(4):438-41.

10. Ahola S, Auranen M, Isohanni P, Niemisalo S, Urho N, Buzkova J, et al. Modified Atkins diet induces subacute selective ragged‐red‐fiber lysis in mitochondrial myopathy patients. EMBO Mol Med. 2016;8(11):1234-47-47.

11. Sofou K, Dahlin M, Hallbook T, Lindefeldt M, Viggedal G, Darin N. Ketogenic diet in pyruvate dehydrogenase complex deficiency: short- and long-term outcomes. J Inherit Metab Dis. 2017;40(2):237-45.

12. Huang L, Li H, Zhong J, Yang L, Chen G, Wang D, et al. Efficacy and Safety of the Ketogenic Diet for Mitochondrial Disease With Epilepsy: A Prospective, Open-labeled, Controlled Study. Front Neurol. 2022;13:880944.

13. Zweers HEE, Kroesen SH, Beerlink G, Buit E, Gerrits K, Dorhout A, et al. Ketogenic diet in adult patients with mitochondrial myopathy. Mol Genet Metab. 2024;143(4):108610.

14. Schoeler NE, Orford M, Vivekananda U, Simpson Z, Van de Bor B, Smith H, et al. K.Vita: a feasibility study of a blend of medium chain triglycerides to manage drug-resistant epilepsy. Brain Commun. 2021;3(4):fcab160.

15. Houghton D, Ng YS, Jackson MA, Stefanetti R, Hynd P, Mac Aogáin M, et al. Phase II Feasibility Study of the Efficacy, Tolerability, and Impact on the Gut Microbiome of a Low-Residue (Fiber) Diet in Adult Patients With Mitochondrial Disease. Gastro Hep Advances. 2022;1(4):666-77.

16. Bastin J, Aubey F, Rötig As, Munnich A, Djouadi F. Activation of Peroxisome Proliferator-Activated Receptor Pathway Stimulates the Mitochondrial Respiratory Chain and Can Correct Deficiencies in Patients’ Cells Lacking Its Components. J Clin Endocrinol Metab. 2008;93(4):1433-41.

17. Douiev L, Sheffer R, Horvath G, Saada A. Bezafibrate Improves Mitochondrial Fission and Function in DNM1L-Deficient Patient Cells. Cells. 2020;9(2).

18. Viscomi C, Bottani E, Civiletto G, Cerutti R, Moggio M, Fagiolari G, et al. In Vivo Correction of COX Deficiency by Activation of the AMPK/PGC-1α Axis. Cell Metab. 2011;14(1):80-90.

19. Yatsuga S, Suomalainen A. Effect of bezafibrate treatment on late-onset mitochondrial myopathy in mice. Hum Mol Genet. 2012;21(3):526-35.

20. Dillon LM, Hida A, Garcia S, Prolla TA, Moraes CT. Long-term bezafibrate treatment improves skin and spleen phenotypes of the mtDNA mutator mouse. PLoS ONE. 2012;7(9):e44335.

21. Lyu J, Zhao Y, Zhang N, Xu X, Zheng R, Yu W, et al. Bezafibrate Rescues Mitochondrial Encephalopathy in Mice via Induction of Daily Torpor and Hypometabolic State. Neurotherapeutics. 2022;19(3):994-1006.

22. Steele H, Gomez-Duran A, Pyle A, Hopton S, Newman J, Stefanetti RJ, et al. Metabolic effects of bezafibrate in mitochondrial disease. EMBO Mol Med. 2020;12(3):e11589.

23. Wang YX, Zhang CL, Yu RT, Cho HK, Nelson MC, Bayuga-Ocampo CR, et al. Regulation of muscle fiber type and running endurance by PPARdelta. PLoS Biol. 2004;2(10):e294.

24. Ito M, Tauscher-Wisniewski S, Smulders RA, Wojtkowski T, Yamada A, Koibuchi A, et al. Single- and multiple-dose safety, tolerability, pharmacokinetic, and pharmacodynamic profiles of ASP0367, or bocidelpar sulfate, a novel modulator of peroxisome proliferator-activated receptor delta in healthy adults: Results from a phase 1 study. Muscle Nerve. 2022;65(1):110-20.

25. Zighan M, Arkadir D, Douiev L, Keller G, Miller C, Saada A. Variable effects of omaveloxolone (RTA408) on primary fibroblasts with mitochondrial defects. Front Mol Biosci. 2022;9:890653.

26. Madsen KL, Buch AE, Cohen BH, Falk MJ, Goldsberry A, Goldstein A, et al. Safety and efficacy of omaveloxolone in patients with mitochondrial myopathy: MOTOR trial. Neurology. 2020;94(7):e687-e98.

27. Baur JA, Pearson KJ, Price NL, Jamieson HA, Lerin C, Kalra A, et al. Resveratrol improves health and survival of mice on a high-calorie diet. Nature. 2006;444(7117):337-42.

28. Lagouge M, Argmann C, Gerhart-Hines Z, Meziane H, Lerin C, Daussin F, et al. Resveratrol Improves Mitochondrial Function and Protects against Metabolic Disease by Activating SIRT1 and PGC-1α. Cell. 2006;127(6):1109-22.

29. Murase T, Haramizu S, Ota N, Hase T. Suppression of the aging-associated decline in physical performance by a combination of resveratrol intake and habitual exercise in senescence-accelerated mice. Biogerontology. 2009;10(4):423-34.

30. Mizuguchi Y, Hatakeyama H, Sueoka K, Tanaka M, Goto Y-i. Low dose resveratrol ameliorates mitochondrial respiratory dysfunction and enhances cellular reprogramming. Mitochondrion. 2017;34:43-8.

31. Løkken N, Khawajazada T, Storgaard JH, Raaschou-Pedersen D, Christensen ME, Hornsyld TM, et al. No effect of resveratrol in patients with mitochondrial myopathy: A cross-over randomized controlled trial. J Inherit Metab Dis. 2021;44(5):1186-98.

32. Golubitzky A, Dan P, Weissman S, Link G, Wikstrom JD, Saada A. Screening for Active Small Molecules in Mitochondrial Complex I Deficient Patient's Fibroblasts, Reveals AICAR as the Most Beneficial Compound. PLoS ONE. 2011;6(10):e26883.

33. Ljubicic V, Miura P, Burt M, Boudreault L, Khogali S, Lunde JA, et al. Chronic AMPK activation evokes the slow, oxidative myogenic program and triggers beneficial adaptations in mdx mouse skeletal muscle. Hum Mol Genet. 2011;20(17):3478-93.

34. Peralta S, Garcia S, Yin HY, Arguello T, Diaz F, Moraes CT. Sustained AMPK activation improves muscle function in a mitochondrial myopathy mouse model by promoting muscle fiber regeneration. Hum Mol Genet. 2016;25(15):3178-91.

35. Hinkle JS, Rivera CN, Vaughan RA. AICAR stimulates mitochondrial biogenesis and BCAA catabolic enzyme expression in C2C12 myotubes. Biochimie. 2022;195:77-85.

36. Nogueira L, Ramirez-Sanchez I, Perkins GA, Murphy A, Taub PR, Ceballos G, et al. (–)-Epicatechin enhances fatigue resistance and oxidative capacity in mouse muscle. J Physiol. 2011;589(18):4615-31.

37. Burgin HJ, Lopez Sanchez MIG, Smith CM, Trounce IA, McKenzie M. Pioglitazone and Deoxyribonucleoside Combination Treatment Increases Mitochondrial Respiratory Capacity in m.3243A>G MELAS Cybrid Cells. Int J Mol Sci. 2020;21(6).

38. Pirinen E, Auranen M, Khan NA, Brilhante V, Urho N, Pessia A, et al. Niacin Cures Systemic NAD(+) Deficiency and Improves Muscle Performance in Adult-Onset Mitochondrial Myopathy. Cell Metab. 2020;31(6):1078-90.e5.

39. Cantó C, Houtkooper RH, Pirinen E, Youn DY, Oosterveer MH, Cen Y, et al. The NAD(+) precursor nicotinamide riboside enhances oxidative metabolism and protects against high-fat diet-induced obesity. Cell Metab. 2012;15(6):838-47.

40. Khan NA, Auranen M, Paetau I, Pirinen E, Euro L, Forsström S, et al. Effective treatment of mitochondrial myopathy by nicotinamide riboside, a vitamin B3. EMBO Mol Med. 2014;6(6):721-31.

41. Cerutti R, Pirinen E, Lamperti C, Marchet S, Sauve AA, Li W, et al. NAD(+)-dependent activation of Sirt1 corrects the phenotype in a mouse model of mitochondrial disease. Cell Metab. 2014;19(6):1042-9.

42. Zhang H, Ryu D, Wu Y, Gariani K, Wang X, Luan P, et al. NAD+ repletion improves mitochondrial and stem cell function and enhances life span in mice. Science. 2016;352(6292):1436-43.

43. Hong Y, Zhang Z, Yangzom T, Chen A, Lundberg BC, Fang EF, et al. The NAD(+) Precursor Nicotinamide Riboside Rescues Mitochondrial Defects and Neuronal Loss in iPSC derived Cortical Organoid of Alpers' Disease. Int J Biol Sci. 2024;20(4):1194-217.

44. Lapatto HAK, Kuusela M, Heikkinen A, Muniandy M, van der Kolk BW, Gopalakrishnan S, et al. Nicotinamide riboside improves muscle mitochondrial biogenesis, satellite cell differentiation, and gut microbiota in a twin study. Sci Adv. 2023;9(2):eadd5163.

45. Lee CF, Caudal A, Abell L, Nagana Gowda GA, Tian R. Targeting NAD(+) Metabolism as Interventions for Mitochondrial Disease. Sci Rep. 2019;9(1):3073.

46. Seo KS, Kim JH, Min KN, Moon JA, Roh TC, Lee MJ, et al. KL1333, a Novel NAD(+) Modulator, Improves Energy Metabolism and Mitochondrial Dysfunction in MELAS Fibroblasts. Front Neurol. 2018;9:552.

47. Pizzamiglio C, Stefanetti RJ, McFarland R, Thomas N, Ransley G, Hugerth M, et al. Optimizing rare disorder trials: a phase 1a/1b randomized study of KL1333 in adults with mitochondrial disease. Brain. 2024;148(1):39-46.

48. van de Weijer T, Phielix E, Bilet L, Williams EG, Ropelle ER, Bierwagen A, et al. Evidence for a direct effect of the NAD+ precursor acipimox on muscle mitochondrial function in humans. Diabetes. 2015;64(4):1193-201.

49. Heitz FD, Erb M, Anklin C, Robay D, Pernet V, Gueven N. Idebenone Protects against Retinal Damage and Loss of Vision in a Mouse Model of Leber’s Hereditary Optic Neuropathy. PLoS ONE. 2012;7(9):e45182.

50. Klopstock T, Yu-Wai-Man P, Dimitriadis K, Rouleau J, Heck S, Bailie M, et al. A randomized placebo-controlled trial of idebenone in Leber's hereditary optic neuropathy. Brain. 2011;134(Pt 9):2677-86.

51. Carelli V, La Morgia C, Valentino ML, Rizzo G, Carbonelli M, De Negri AM, et al. Idebenone treatment in Leber's hereditary optic neuropathy. Brain. 2011;134(Pt 9):e188.

52. Klopstock T, Metz G, Yu-Wai-Man P, Büchner B, Gallenmüller C, Bailie M, et al. Persistence of the treatment effect of idebenone in Leber’s hereditary optic neuropathy. Brain. 2013;136(2):e230-e.

53. Catarino CB, von Livonius B, Priglinger C, Banik R, Matloob S, Tamhankar MA, et al. Real-World Clinical Experience With Idebenone in the Treatment of Leber Hereditary Optic Neuropathy. J Neuroophthalmol. 2020;40(4):558-65.

54. van Everdingen JAM, Pott JWR, Bauer NJC, Krijnen AM, Lushchyk T, Wubbels RJ. Clinical outcomes of treatment with idebenone in Leber's hereditary optic neuropathy in the Netherlands: A national cohort study. Acta Ophthalmol. 2022;100(6):700-6.

55. Valentin K, Georgi T, Riedl R, Aminfar H, Singer C, Klopstock T, et al. Idebenone Treatment in Patients with OPA1-Dominant Optic Atrophy: A Prospective Phase 2 Trial. Neuroophthalmology. 2023;47(5-6):237-47.

56. Yu-Wai-Man P, Carelli V, Newman NJ, Silva MJ, Linden A, Van Stavern G, et al. Therapeutic benefit of idebenone in patients with Leber hereditary optic neuropathy: The LEROS nonrandomized controlled trial. Cell Rep Med. 2024;5(3):101437.

57. Newman NJ, Biousse V, Yu-Wai-Man P, Carelli V, Vignal-Clermont C, Montestruc F, et al. Meta-analysis of treatment outcomes for patients with m.11778G>A MT-ND4 Leber Hereditary Optic Neuropathy. Surv Ophthalmol. 2024.

58. Shrader WD, Amagata A, Barnes A, Enns GM, Hinman A, Jankowski O, et al. alpha-Tocotrienol quinone modulates oxidative stress response and the biochemistry of aging. Bioorg Med Chem Lett. 2011;21(12):3693-8.

59. Kahn-Kirby AH, Amagata A, Maeder CI, Mei JJ, Sideris S, Kosaka Y, et al. Targeting ferroptosis: A novel therapeutic strategy for the treatment of mitochondrial disease-related epilepsy. PLoS ONE. 2019;14(3):e0214250.

60. Kayser EB, Chen Y, Mulholland M, Truong V, James K, Hanaford A, et al. Evaluating the efficacy of vatiquinone in preclinical models of mitochondrial disease. Res Sq. 2024.

61. Enns GM, Kinsman SL, Perlman SL, Spicer KM, Abdenur JE, Cohen BH, et al. Initial experience in the treatment of inherited mitochondrial disease with EPI-743. Mol Genet Metab. 2012;105(1):91-102.

62. Martinelli D, Catteruccia M, Piemonte F, Pastore A, Tozzi G, Dionisi-Vici C, et al. EPI-743 reverses the progression of the pediatric mitochondrial disease--genetically defined Leigh Syndrome. Mol Genet Metab. 2012;107(3):383-8.

63. Blankenberg FG, Kinsman SL, Cohen BH, Goris ML, Spicer KM, Perlman SL, et al. Brain uptake of Tc99m-HMPAO correlates with clinical response to the novel redox modulating agent EPI-743 in patients with mitochondrial disease. Mol Genet Metab. 2012;107(4):690-9.

64. Sadun AA, Chicani CF, Ross-Cisneros FN, Barboni P, Thoolen M, Shrader WD, et al. Effect of EPI-743 on the clinical course of the mitochondrial disease Leber hereditary optic neuropathy. Arch Neurol. 2012;69(3):331-8.

65. Pastore A, Petrillo S, Tozzi G, Carrozzo R, Martinelli D, Dionisi-Vici C, et al. Glutathione: A redox signature in monitoring EPI-743 therapy in children with mitochondrial encephalomyopathies. Mol Genet Metab. 2013;109(2):208-14.

66. Enns GM, Cohen BH. Clinical Trials in Mitochondrial Disease:An Update on EPI-743 and RP103. J Inborn Errors Metab Screen. 2017;5:2326409817733013.

67. de Haas R, Das D, Garanto A, Renkema HG, Greupink R, van den Broek P, et al. Therapeutic effects of the mitochondrial ROS-redox modulator KH176 in a mammalian model of Leigh Disease. Sci Rep. 2017;7(1):11733.

68. Beyrath J, Pellegrini M, Renkema H, Houben L, Pecheritsyna S, van Zandvoort P, et al. KH176 Safeguards Mitochondrial Diseased Cells from Redox Stress-Induced Cell Death by Interacting with the Thioredoxin System/Peroxiredoxin Enzyme Machinery. Sci. 2018;8(1):6577.

69. Klein Gunnewiek TM, Verboven AHA, Pelgrim I, Hogeweg M, Schoenmaker C, Renkema H, et al. Sonlicromanol improves neuronal network dysfunction and transcriptome changes linked to m.3243A&gt;G heteroplasmy in iPSC-derived neurons. Stem Cell Reports. 2021;16(9):2197-212.

70. Janssen MCH, Koene S, de Laat P, Hemelaar P, Pickkers P, Spaans E, et al. The KHENERGY Study: Safety and Efficacy of KH176 in Mitochondrial m.3243A>G Spectrum Disorders. Clin Pharmacol Ther. 2019;105(1):101-11.

71. Smeitink J, van Es J, Bosman B, Janssen MCH, Klopstock T, Gorman G, et al. Phase 2b program with sonlicromanol in patients with mitochondrial disease due to m.3243A>G mutation. Brain. 2024.

72. Guha S, Konkwo C, Lavorato M, Mathew ND, Peng M, Ostrovsky J, et al. Pre-clinical evaluation of cysteamine bitartrate as a therapeutic agent for mitochondrial respiratory chain disease. Hum Mol Genet. 2019;28(11):1837-52.

73. Haroon S, Yoon H, Seiler C, Osei-Frimpong B, He J, Nair RM, et al. N-acetylcysteine and cysteamine bitartrate prevent azide-induced neuromuscular decompensation by restoring glutathione balance in two novel surf1-/- zebrafish deletion models of Leigh syndrome. Hum Mol Genet. 2023;32(12):1988-2004.

74. Douiev L, Soiferman D, Alban C, Saada A. The Effects of Ascorbate, N-Acetylcysteine, and Resveratrol on Fibroblasts from Patients with Mitochondrial Disorders. J Clin Med. 2016;6(1).

75. Dogan SA, Cerutti R, Benincá C, Brea-Calvo G, Jacobs HT, Zeviani M, et al. Perturbed Redox Signaling Exacerbates a Mitochondrial Myopathy. Cell Metab. 2018;28(5):764-75.e5.

76. Polyak E, Ostrovsky J, Peng M, Dingley SD, Tsukikawa M, Kwon YJ, et al. N-acetylcysteine and vitamin E rescue animal longevity and cellular oxidative stress in pre-clinical models of mitochondrial complex I disease. Mol Genet Metab. 2018;123(4):449-62.

77. Guha S, Mathew ND, Konkwo C, Ostrovsky J, Kwon YJ, Polyak E, et al. Combinatorial glucose, nicotinic acid and N-acetylcysteine therapy has synergistic effect in preclinical C. elegans and zebrafish models of mitochondrial complex I disease. Hum Mol Genet. 2021;30(7):536-51.

78. Kwok WT, Kwak HA, Andreazza AC. N-acetylcysteine modulates rotenone-induced mitochondrial Complex I dysfunction in THP-1 cells. Mitochondrion. 2023;72:1-10.

79. Viscomi C, Burlina AB, Dweikat I, Savoiardo M, Lamperti C, Hildebrandt T, et al. Combined treatment with oral metronidazole and N-acetylcysteine is effective in ethylmalonic encephalopathy. Nat Med. 2010;16(8):869-71.

80. Kılıç M, Dedeoğlu Ö, Göçmen R, Kesici S, Yüksel D. Successful treatment of a patient with ethylmalonic encephalopathy by intravenous N-acetylcysteine. Metab Brain Dis. 2017;32(2):293-6.

81. Moss HG, Brown TR, Wiest DB, Jenkins DD. N-Acetylcysteine rapidly replenishes central nervous system glutathione measured via magnetic resonance spectroscopy in human neonates with hypoxic-ischemic encephalopathy. J Cereb Blood Flow Metab. 2018;38(6):950-8.

82. Shayota BJ, Soler-Alfonso C, Bekheirnia MR, Mizerik E, Boyer SW, Xiao R, et al. Case report and novel treatment of an autosomal recessive Leigh syndrome caused by short-chain enoyl-CoA hydratase deficiency. Am J Med Genet A. 2019;179(5):803-7.

83. Kitzler TM, Gupta IR, Osterman B, Poulin C, Trakadis Y, Waters PJ, et al. Acute and Chronic Management in an Atypical Case of Ethylmalonic Encephalopathy. JIMD Rep. 2019;45:57-63.

84. Koga Y, Akita Y, Nishioka J, Yatsuga S, Povalko N, Tanabe Y, et al. L-arginine improves the symptoms of strokelike episodes in MELAS. Neurology. 2005;64(4):710-2.

85. Koga Y, Akita Y, Junko N, Yatsuga S, Povalko N, Fukiyama R, et al. Endothelial dysfunction in MELAS improved by l-arginine supplementation. Neurology. 2006;66(11):1766-9.

86. El-Hattab AW, Hsu JW, Emrick LT, Wong L-JC, Craigen WJ, Jahoor F, et al. Restoration of impaired nitric oxide production in MELAS syndrome with citrulline and arginine supplementation. Mol Genet Metab. 2012;105(4):607-14.

87. Rodan LH, Wells GD, Banks L, Thompson S, Schneiderman JE, Tein I. L-Arginine Affects Aerobic Capacity and Muscle Metabolism in MELAS (Mitochondrial Encephalomyopathy, Lactic Acidosis and Stroke-Like Episodes) Syndrome. PLoS ONE. 2015;10(5):e0127066.

88. El-Hattab AW, Emrick LT, Hsu JW, Chanprasert S, Almannai M, Craigen WJ, et al. Impaired nitric oxide production in children with MELAS syndrome and the effect of arginine and citrulline supplementation. Mol Genet Metab. 2016;117(4):407-12.

89. Koga Y, Povalko N, Inoue E, Nakamura H, Ishii A, Suzuki Y, et al. Therapeutic regimen of L-arginine for MELAS: 9-year, prospective, multicenter, clinical research. J Neurol. 2018;265(12):2861-74.

90. Ganetzky RD, Falk MJ. 8-year retrospective analysis of intravenous arginine therapy for acute metabolic strokes in pediatric mitochondrial disease. Mol Genet Metab. 2018;123(3):301-8.

91. Ikawa M, Povalko N, Koga Y. Arginine therapy in mitochondrial myopathy, encephalopathy, lactic acidosis, and stroke-like episodes. Current Opinion in Clinical Nutrition & Metabolic Care. 2020;23(1):17-22.

92. Al Jasmi F, Al Zaabi N, Al-Thihli K, Al Teneiji AM, Hertecant J, El-Hattab AW. Endothelial Dysfunction and the Effect of Arginine and Citrulline Supplementation in Children and Adolescents With Mitochondrial Diseases. J Cent Nerv Syst Dis. 2020;12:1179573520909377.

93. Rodan LH, Poublanc J, Fisher JA, Sobczyk O, Mikulis DJ, Tein I. L-arginine effects on cerebrovascular reactivity, perfusion and neurovascular coupling in MELAS (mitochondrial encephalomyopathy with lactic acidosis and stroke-like episodes) syndrome. PLoS ONE. 2020;15(9):e0238224.

94. Stefanetti RJ, Ng YS, Errington L, Blain AP, McFarland R, Gorman GS. l-Arginine in Mitochondrial Encephalopathy, Lactic Acidosis, and Stroke-like Episodes: A Systematic Review. Neurology. 2022;98(23):e2318-e28.

95. Correia SS, Iyengar RR, Germano P, Tang K, Bernier SG, Schwartzkopf CD, et al. The CNS-Penetrant Soluble Guanylate Cyclase Stimulator CY6463 Reveals its Therapeutic Potential in Neurodegenerative Diseases. Front Pharmacol. 2021;12:656561.

96. Johnson SC, Yanos ME, Kayser EB, Quintana A, Sangesland M, Castanza A, et al. mTOR inhibition alleviates mitochondrial disease in a mouse model of Leigh syndrome. Science. 2013;342(6165):1524-8.

97. Wang A, Mouser J, Pitt J, Promislow D, Kaeberlein M. Rapamycin enhances survival in a Drosophila model of mitochondrial disease. Oncotarget. 2016;7(49).

98. Zheng X, Boyer L, Jin M, Kim Y, Fan W, Bardy C, et al. Alleviation of neuronal energy deficiency by mTOR inhibition as a treatment for mitochondria-related neurodegeneration. eLife. 2016;5:e13378.

99. Khan NA, Nikkanen J, Yatsuga S, Jackson C, Wang L, Pradhan S, et al. mTORC1 Regulates Mitochondrial Integrated Stress Response and Mitochondrial Myopathy Progression. Cell Metab. 2017;26(2):419-28.e5.

100. Civiletto G, Dogan SA, Cerutti R, Fagiolari G, Moggio M, Lamperti C, et al. Rapamycin rescues mitochondrial myopathy via coordinated activation of autophagy and lysosomal biogenesis. EMBO Mol Med. 2018;10(11):e8799.

101. Johnson SC, Martinez F, Bitto A, Gonzalez B, Tazaerslan C, Cohen C, et al. mTOR inhibitors may benefit kidney transplant recipients with mitochondrial diseases. Kidney Int. 2019;95(2):455-66.

102. Barriocanal-Casado E, Hidalgo-Gutiérrez A, Raimundo N, González-García P, Acuña-Castroviejo D, Escames G, et al. Rapamycin administration is not a valid therapeutic strategy for every case of mitochondrial disease. EBioMedicine. 2019;42:511-23.

103. Martin-Perez M, Grillo AS, Ito TK, Valente AS, Han J, Entwisle SW, et al. PKC downregulation upon rapamycin treatment attenuates mitochondrial disease. Nat Metab. 2020;2(12):1472-81.

104. Stokes JC, Bornstein RL, James K, Park KY, Spencer KA, Vo K, et al. Leukocytes mediate disease pathogenesis in the Ndufs4(KO) mouse model of Leigh syndrome. JCI Insight. 2022;7(5).

105. Sage-Schwaede A, Engelstad K, Salazar R, Curcio A, Khandji A, Garvin JH, Jr., et al. Exploring mTOR inhibition as treatment for mitochondrial disease. Ann Clin Transl Neurol. 2019;6(9):1877-81.

106. Szeto HH, Liu S, Soong Y, Wu D, Darrah SF, Cheng FY, et al. Mitochondria-targeted peptide accelerates ATP recovery and reduces ischemic kidney injury. J Am Soc Nephrol. 2011;22(6):1041-52.

107. Birk AV, Liu S, Soong Y, Mills W, Singh P, Warren JD, et al. The Mitochondrial-Targeted Compound SS-31 Re-Energizes Ischemic Mitochondria by Interacting with Cardiolipin. J Am Soc Nephrol. 2013;24(8):1250-61.

108. Siegel MP, Kruse SE, Percival JM, Goh J, White CC, Hopkins HC, et al. Mitochondrial-targeted peptide rapidly improves mitochondrial energetics and skeletal muscle performance in aged mice. Aging Cell. 2013;12(5):763-71.

109. Brown DA, Hale SL, Baines CP, del Rio CL, Hamlin RL, Yueyama Y, et al. Reduction of early reperfusion injury with the mitochondria-targeting peptide bendavia. J Cardiovasc Pharmacol Ther. 2014;19(1):121-32.

110. Zhao W, Xu Z, Cao J, Fu Q, Wu Y, Zhang X, et al. Elamipretide (SS-31) improves mitochondrial dysfunction, synaptic and memory impairment induced by lipopolysaccharide in mice. J Neuroinflammation. 2019;16(1):230.

111. Chatfield KC, Sparagna GC, Chau S, Phillips EK, Ambardekar AV, Aftab M, et al. Elamipretide Improves Mitochondrial Function in the Failing Human Heart. JACC Basic Transl Sci. 2019;4(2):147-57.

112. Allen ME, Pennington ER, Perry JB, Dadoo S, Makrecka-Kuka M, Dambrova M, et al. The cardiolipin-binding peptide elamipretide mitigates fragmentation of cristae networks following cardiac ischemia reperfusion in rats. Commun Biol. 2020;3(1):389.

113. Roshanravan B, Liu SZ, Ali AS, Shankland EG, Goss C, Amory JK, et al. In vivo mitochondrial ATP production is improved in older adult skeletal muscle after a single dose of elamipretide in a randomized trial. PLoS ONE. 2021;16(7):e0253849.

114. Campbell MD, Samuelson AT, Chiao YA, Sweetwyne MT, Ladiges WC, Rabinovitch PS, et al. Intermittent treatment with elamipretide preserves exercise tolerance in aged female mice. Geroscience. 2023;45(4):2245-55.

115. Russo S, De Rasmo D, Rossi R, Signorile A, Lobasso S. SS-31 treatment ameliorates cardiac mitochondrial morphology and defective mitophagy in a murine model of Barth syndrome. Sci Rep. 2024;14(1):13655.

116. Karaa A, Haas R, Goldstein A, Vockley J, Weaver WD, Cohen BH. Randomized dose-escalation trial of elamipretide in adults with primary mitochondrial myopathy. Neurology. 2018;90(14):e1212-e21.

117. Karaa A, Haas R, Goldstein A, Vockley J, Cohen BH. A randomized crossover trial of elamipretide in adults with primary mitochondrial myopathy. J Cachexia Sarcopenia Muscle. 2020;11(4):909-18.

118. Oates PJ, Brown DA, Vernon HJ, Gangoiti JA, Barshop BA. Metabolomic biomarkers from patients with Barth syndrome treated with elamipretide: insights from the TAZPOWER study. medRxiv. 2020:2020.11.20.20235580.

119. Reid Thompson W, Hornby B, Manuel R, Bradley E, Laux J, Carr J, et al. A phase 2/3 randomized clinical trial followed by an open-label extension to evaluate the effectiveness of elamipretide in Barth syndrome, a genetic disorder of mitochondrial cardiolipin metabolism. Genet Med. 2021;23(3):471-8.

120. Hornby B, Thompson WR, Almuqbil M, Manuel R, Abbruscato A, Carr J, et al. Natural history comparison study to assess the efficacy of elamipretide in patients with Barth syndrome. Orphanet J Rare Dis. 2022;17(1):336.

121. Karaa A, Bertini E, Carelli V, Cohen BH, Enns GM, Falk MJ, et al. Efficacy and Safety of Elamipretide in Individuals With Primary Mitochondrial Myopathy: The MMPOWER-3 Randomized Clinical Trial. Neurology. 2023;101(3):e238-e52.

122. Koenig MK, Russo SN, McBride KL, Bjornsson HT, Gunnarsdottir BB, Goldstein A, et al. Use of Elamipretide in patients assigned treatment in the compassionate use program: Case series in pediatric patients with rare orphan diseases. JIMD Rep. 2023;64(1):65-70.

123. Thompson WR, Manuel R, Abbruscato A, Carr J, Campbell J, Hornby B, et al. Long-term efficacy and safety of elamipretide in patients with Barth syndrome: 168-week open-label extension results of TAZPOWER. Genet Med. 2024;26(7):101138.

124. Karanjia R, Sadun AA. Elamipretide Topical Ophthalmic Solution for the Treatment of Subjects with Leber Hereditary Optic Neuropathy: A Randomized Trial. Ophthalmology. 2024;131(4):422-33.

125. Bulst S, Holinski-Feder E, Payne B, Abicht A, Krause S, Lochmüller H, et al. In vitro supplementation with deoxynucleoside monophosphates rescues mitochondrial DNA depletion. Mol Genet Metab. 2012;107(1):95-103.

126. Cámara Y, González-Vioque E, Scarpelli M, Torres-Torronteras J, Caballero A, Hirano M, et al. Administration of deoxyribonucleosides or inhibition of their catabolism as a pharmacological approach for mitochondrial DNA depletion syndrome. Hum Mol Genet. 2013;23(9):2459-67.

127. Garone C, Garcia‐Diaz B, Emmanuele V, Lopez LC, Tadesse S, Akman HO, et al. Deoxypyrimidine monophosphate bypass therapy for thymidine kinase 2 deficiency. EMBO Mol Med. 2014;6(8):1016-27.

128. Lopez-Gomez C, Levy RJ, Sanchez-Quintero MJ, Juanola-Falgarona M, Barca E, Garcia-Diaz B, et al. Deoxycytidine and Deoxythymidine Treatment for Thymidine Kinase 2 Deficiency. Ann Neurol. 2017;81(5):641-52.

129. Dombi E, Marinaki T, Spingardi P, Millar V, Hadjichristou N, Carver J, et al. Nucleoside supplements as treatments for mitochondrial DNA depletion syndrome. Front Cell Dev Biol. 2024;12:1260496.

130. Domínguez-González C, Madruga-Garrido M, Mavillard F, Garone C, Aguirre-Rodríguez FJ, Donati MA, et al. Deoxynucleoside Therapy for Thymidine Kinase 2–Deficient Myopathy. Ann Neurol. 2019;86(2):293-303.

131. Pekeles H, Berrahmoune S, Dassi C, Cheung ACT, Gagnon T, Waters PJ, et al. Safety and efficacy of deoxycytidine/deoxythymidine combination therapy in POLG-related disorders: 6-month interim results of an open-label, single arm, phase 2 trial. EClinicalMedicine. 2024;74:102740.

132. Bermejo-Guerrero L, Hernández-Voth A, Serrano-Lorenzo P, Blázquez A, Martin-Jimenez P, Martin MA, et al. Remarkable clinical improvement with oral nucleoside treatment in a patient with adult-onset TK2 deficiency: A case report. Mitochondrion. 2024;76:101879.

133. Moran NF, Bain MD, Muqit MMK, Bax BE. Carrier erythrocyte entrapped thymidine phosphorylase therapy for MNGIE. Neurology. 2008;71(9):686-8.

134. Bax BE, Bain MD, Scarpelli M, Filosto M, Tonin P, Moran N. Clinical and biochemical improvements in a patient with MNGIE following enzyme replacement. Neurology. 2013;81(14):1269-71.

135. Wong LJ, Naviaux RK, Brunetti-Pierri N, Zhang Q, Schmitt ES, Truong C, et al. Molecular and clinical genetics of mitochondrial diseases due to POLG mutations. Hum Mutat. 2008;29(9):E150-72.

136. El-Hattab AW WJ, Dai H, et al. MPV17-Related Mitochondrial DNA Maintenance Defect. 2012 1993-2024. In: GeneReviews® [Internet] [Internet]. Seattle (WA): University of Washington, Seattle. Available from: <https://www.ncbi.nlm.nih.gov/books/NBK92947/>.

137. Grabhorn E, Tsiakas K, Herden U, Fischer L, Freisinger P, Marquardt T, et al. Long-term outcomes after liver transplantation for deoxyguanosine kinase deficiency: a single-center experience and a review of the literature. Liver Transpl. 2014;20(4):464-72.

138. Parikh S, Karaa A, Goldstein A, Ng YS, Gorman G, Feigenbaum A, et al. Solid organ transplantation in primary mitochondrial disease: Proceed with caution. Mol Genet Metab. 2016;118(3):178-84.

139. Dionisi-Vici C, Diodato D, Torre G, Picca S, Pariante R, Giuseppe Picardo S, et al. Liver transplant in ethylmalonic encephalopathy: a new treatment for an otherwise fatal disease. Brain. 2016;139(4):1045-51.

140. Tam A, AlDhaheri NS, Mysore K, Tessier ME, Goss J, Fernandez LA, et al. Improved clinical outcome following liver transplant in patients with ethylmalonic encephalopathy. Am J Med Genet A. 2019;179(6):1015-9.

141. Shimura M, Kuranobu N, Ogawa-Tominaga M, Akiyama N, Sugiyama Y, Ebihara T, et al. Clinical and molecular basis of hepatocerebral mitochondrial DNA depletion syndrome in Japan: evaluation of outcomes after liver transplantation. Orphanet J Rare Dis. 2020;15(1):169.

142. Uchida H, Sakamoto S, Shimizu S, Yanagi Y, Fukuda A, Horikawa R, et al. Outcomes of liver transplantation for mitochondrial respiratory chain disorder in children. Pediatr Transplant. 2021;25(8):e14091.

143. Olivieri G, Martinelli D, Longo D, Grimaldi C, Liccardo D, Di Meo I, et al. Ethylmalonic encephalopathy and liver transplantation: long-term outcome of the first treated patient. Orphanet J Rare Dis. 2021;16(1):229.

144. Jankowska I, Czubkowski P, Rokicki D, Lipiński P, Piekutowska-Abramczuk D, Ciara E, et al. Acute liver failure due to DGUOK deficiency-is liver transplantation justified? Clin Res Hepatol Gastroenterol. 2021;45(1):101408.

145. Hirano M, Carelli V, De Giorgio R, Pironi L, Accarino A, Cenacchi G, et al. Mitochondrial neurogastrointestinal encephalomyopathy (MNGIE): Position paper on diagnosis, prognosis, and treatment by the MNGIE International Network. J Inherit Metab Dis. 2021;44(2):376-87.

146. Islam MN, Das SR, Emin MT, Wei M, Sun L, Westphalen K, et al. Mitochondrial transfer from bone-marrow-derived stromal cells to pulmonary alveoli protects against acute lung injury. Nat Med. 2012;18(5):759-65.

147. Ahmad T, Mukherjee S, Pattnaik B, Kumar M, Singh S, Kumar M, et al. Miro1 regulates intercellular mitochondrial transport & enhances mesenchymal stem cell rescue efficacy. The EMBO Journal. 2014;33(9):994-1010-.

148. Jacoby E, Ben Yakir-Blumkin M, Blumenfeld-Kan S, Brody Y, Meir A, Melamed-Book N, et al. Mitochondrial augmentation of CD34(+) cells from healthy donors and patients with mitochondrial DNA disorders confers functional benefit. NPJ Regen Med. 2021;6(1):58.

149. Nakai R, Varnum S, Field RL, Shi H, Giwa R, Jia W, et al. Mitochondria transfer-based therapies reduce the morbidity and mortality of Leigh syndrome. Nat Metab. 2024.

150. Jacoby E, Blumkin M, Anikster Y, Varda-Bloom N, Pansheen J, Bar Yoseph O, et al. First-in-Human Mitochondrial Augmentation of Hematopoietic Stem Cells in Pearson Syndrome. Blood. 2018;132(Supplement 1):1024-.

151. Yosef OB, Jacoby E, Gruber N, Varda-Bloom N, Azaria E, Eisenstein E, et al. Promising Results for Kearns-Sayre Syndrome of First in Man Treatment by Mitochondrial Augmentation Therapy (457). Neurology. 2020;94(15_supplement):457.

152. Jacoby E, Bar-Yosef O, Gruber N, Lahav E, Varda-Bloom N, Bolkier Y, et al. Mitochondrial augmentation of hematopoietic stem cells in children with single large-scale mitochondrial DNA deletion syndromes. Sci Transl Med. 2022;14(676):eabo3724.

153. van Tienen F, Zelissen R, Timmer E, van Gisbergen M, Lindsey P, Quattrocelli M, et al. Healthy, mtDNA-mutation free mesoangioblasts from mtDNA patients qualify for autologous therapy. Stem Cell Res Ther. 2019;10(1):405.

154. Lavorato M, Nakamaru-Ogiso E, Mathew ND, Herman E, Shah N, Haroon S, et al. Dichloroacetate improves mitochondrial function, physiology, and morphology in FBXL4 disease models. JCI Insight. 2022;7(16).

155. Broxton CN, Kaur P, Lavorato M, Ganesh S, Xiao R, Mathew ND, et al. Dichloroacetate and thiamine improve survival and mitochondrial stress in a C. elegans model of dihydrolipoamide dehydrogenase deficiency. JCI Insight. 2022;7(20).

156. Stacpoole PW, Kerr DS, Barnes C, Bunch ST, Carney PR, Fennell EM, et al. Controlled clinical trial of dichloroacetate for treatment of congenital lactic acidosis in children. Pediatrics. 2006;117(5):1519-31.

157. Duncan GE, Perkins LA, Theriaque DW, Neiberger RE, Stacpoole PW. Dichloroacetate therapy attenuates the blood lactate response to submaximal exercise in patients with defects in mitochondrial energy metabolism. J Clin Endocrinol Metab. 2004;89(4):1733-8.

158. Berendzen K, Theriaque DW, Shuster J, Stacpoole PW. Therapeutic potential of dichloroacetate for pyruvate dehydrogenase complex deficiency. Mitochondrion. 2006;6(3):126-35.

159. Kaufmann P, Engelstad K, Wei Y, Jhung S, Sano MC, Shungu DC, et al. Dichloroacetate causes toxic neuropathy in MELAS: a randomized, controlled clinical trial. Neurology. 2006;66(3):324-30.

160. Ferriero R, Manco G, Lamantea E, Nusco E, Ferrante MI, Sordino P, et al. Phenylbutyrate therapy for pyruvate dehydrogenase complex deficiency and lactic acidosis. Sci Transl Med. 2013;5(175):175ra31.

161. Ferriero R, Boutron A, Brivet M, Kerr D, Morava E, Rodenburg RJ, et al. Phenylbutyrate increases pyruvate dehydrogenase complex activity in cells harboring a variety of defects. Ann Clin Transl Neurol. 2014;1(7):462-70.

162. Adant I, Bird M, Decru B, Windmolders P, Wallays M, de Witte P, et al. Pyruvate and uridine rescue the metabolic profile of OXPHOS dysfunction. Mol Metab. 2022;63:101537.

163. Li M, Zhou S, Chen C, Ma L, Luo D, Tian X, et al. Therapeutic potential of pyruvate therapy for patients with mitochondrial diseases: a systematic review. Ther Adv Endocrinol Metab. 2020;11:2042018820938240.

164. Suzuki T, Suzuki T, Wada T, Saigo K, Watanabe K. Taurine as a constituent of mitochondrial tRNAs: new insights into the functions of taurine and human mitochondrial diseases. The EMBO Journal. 2002;21(23):6581-9.

165. Jong CJ, Sandal P, Schaffer SW. The Role of Taurine in Mitochondria Health: More Than Just an Antioxidant. Molecules. 2021;26(16).

166. Homma K, Toda E, Osada H, Nagai N, Era T, Tsubota K, et al. Taurine rescues mitochondria-related metabolic impairments in the patient-derived induced pluripotent stem cells and epithelial-mesenchymal transition in the retinal pigment epithelium. Redox Biol. 2021;41:101921.

167. Ohsawa Y, Hagiwara H, Nishimatsu S-i, Hirakawa A, Kamimura N, Ohtsubo H, et al. Taurine supplementation for prevention of stroke-like episodes in MELAS: a multicentre, open-label, 52-week phase III trial. J Neurol Neurosurg Psychiatry. 2019;90(5):529-36.

168. Kranrod J, Konkel A, Valencia R, Darwesh AM, Fischer R, Schunck WH, et al. Cardioprotective properties of OMT-28, a synthetic analog of omega-3 epoxyeicosanoids. J Biol Chem. 2024;300(6):107372.

169. Jain IH, Zazzeron L, Goli R, Alexa K, Schatzman-Bone S, Dhillon H, et al. Hypoxia as a therapy for mitochondrial disease. Science. 2016;352(6281):54-61.

170. Ferrari M, Jain IH, Goldberger O, Rezoagli E, Thoonen R, Cheng KH, et al. Hypoxia treatment reverses neurodegenerative disease in a mouse model of Leigh syndrome. Proc Natl Acad Sci U S A. 2017;114(21):E4241-e50.

171. Jain IH, Zazzeron L, Goldberger O, Marutani E, Wojtkiewicz GR, Ast T, et al. Leigh Syndrome Mouse Model Can Be Rescued by Interventions that Normalize Brain Hyperoxia, but Not HIF Activation. Cell Metab. 2019;30(4):824-32.e3.
